# Oligodendrocyte Piezo2 is a regulator of age-dependent myelin integrity and dysregulated in multiple sclerosis

**DOI:** 10.1038/s42003-026-10530-3

**Published:** 2026-06-20

**Authors:** Julia Dyckow-Schubart, Amelie M. Rabitsch, Celine Geywitz, Christina Mayer, Celia Lerma-Martin, Natalie Ludwig, Matthew Smith, Heidrun Potschka, Peter A. Calabresi, Klaus-Armin Nave, Manuel A. Friese, Wiebke Möbius, Lucas Schirmer

**Affiliations:** 1 Division of Neuroimmunology, Department of Neurology, Medical Faculty Mannheim, Heidelberg University, Mannheim, Germany; 2 Interdisciplinary Center for Neurosciences (IZN), Heidelberg University, Heidelberg, Germany; 3 Institute of Neuroimmunology and Multiple Sclerosis, Center for Molecular Neurobiology Hamburg, University Medical Center Hamburg-Eppendorf (UKE), Hamburg, Germany; 4 Departments of Neurology and Neuroscience, Johns Hopkins University School of Medicine, Baltimore, MD USA; 5 Institute of Pharmacology, Toxicology and Pharmacy, Ludwig-Maximilians-Universität, Munich, Germany; 6 Department of Neurogenetics, Max Planck Institute for Multidisciplinary Sciences, Göttingen, Germany; 7 Mannheim Center for Translational Neuroscience (MCTN) and Institute for Innate Immunoscience (MI3), Medical Faculty Mannheim, Heidelberg University, Mannheim, Germany

**Keywords:** Ion channels in the nervous system, Neuroimmunology

## Abstract

Studies on the mechanosensitive ion channel Piezo2 largely focus on its role in the peripheral nervous system, particularly in touch and pain sensation. Here, we investigate Piezo2 function in the anterior visual pathway of the central nervous system with a focus on oligodendrocyte (OL) biology and myelin integrity. Using single-nucleus RNA sequencing, we identify *Piezo2* expression in late differentiated OLs of the murine optic nerve, with minor expression in retinal ganglion cells. OL-specific *Piezo2* deficiency results in age-dependent motor impairment and selective disruption of myelin compaction in small-caliber optic nerve axons, a fiber population known to be particularly vulnerable in demyelinating disease. Differential gene expression analysis further indicates that Piezo2 regulates myelin compaction and white matter integrity in mature OLs. Consistent with these findings, OL-encoded *PIEZO2* expression is reduced in optic nerve lesion areas from multiple sclerosis patients, highlighting a convergent mechanism of small-caliber fiber vulnerability. Together, these data identify Piezo2 as an age-related regulator of OL function and myelin integrity, with potential relevance for preserving white matter structure in multiple sclerosis.

## Abstract (web summary)

Oligodendroglial Piezo2 is shown to regulate myelin compaction in small optic nerve axons. Its downregulation in multiple sclerosis suggests Piezo2 as a therapeutic target to support white matter integrity in chronic inflammation.

## Introduction

Piezo proteins are a class of mechanically activated cation channels in vertebrates, which consists of two members, Piezo1 (Fam38A) and Piezo2 (Fam38B)^1,2^. Activation of these pore-forming transmembrane channels induces non-selective cation currents, leading to membrane depolarization and calcium-dependent signaling pathways^3^. So far, most studies focused on the slowly-adapting channel Piezo1, which is associated with vascular development^1,4^, regulation of cell density and proliferation^5,6^, and mediating shear stress in different compartments and cell types^7–11^. In the nervous system, for example, Piezo1 has been reported to provide oligodendrocyte precursor cells (OPCs) with information about mechanical properties of their extracellular environment (ECM)^12^.

Piezo2, on the other hand, adapts rapidly and is known to be expressed in dorsal root ganglia and in the skin, conferring proprioception, touch and pain sensation^13–16^. Its function has been studied in the peripheral nervous system (PNS), where it has been reported to play a role in Schwann cell (SC) myelination^17^. Although one study reported *Piezo2* gene expression in parts of the central nervous system (CNS) including the optic nerve (ON), brain and corpus callosum by using conventional PCR, focus was not on oligodendrocytes (OLs)^18^, hence, its cellular distribution and functional role remain poorly characterized. To address this gap, we aimed to investigate its expression pattern and function in the CNS with a focus on the anterior visual system, consisting of ON white matter (WM) and retinal gray matter with its projection neurons, the retinal ganglion cells (RGCs).

Of note, the ON represents one of the CNS regions most susceptible to demyelination and inflammation in multiple sclerosis (MS), the most prevalent chronic inflammatory CNS disease in humans, leading to progressive neuroaxonal damage^19^ and associated with alterations of ECM stiffness^20–22^. OLs, the myelinating cells of the CNS that ensheath axons with concentric tightly packed layers of myelin, are essential for fast saltatory conduction and are key disease targets in MS^23^. Given that OLs contribute to the CNS extracellular environment^24^ and are directly influenced by the ECM in their function^24–26^, and that myelination can be modulated by axonal electrical activity through OL calcium transients^27,28^, we hypothesized that OL-intrinsic Piezo2-mediated mechanosensation plays roles in myelination and myelin integrity, which becomes dysregulated in diseases like MS^29^. Importantly, small-caliber axons are known to be particularly vulnerable to demyelination and degeneration in MS^30,31^, yet the molecular mechanisms underlying this selective susceptibility remain poorly understood. We therefore specifically considered whether mechanosensitive pathways in OLs may differentially contribute to the maintenance of small-caliber fiber integrity.

In this study, we used single-nucleus RNA-sequencing (snRNA-seq) and in situ hybridization (ISH) to study *Piezo1* and *Piezo2* expression in the distinct cells of the anterior visual system including mouse ON and retina. As *Piezo2* was mainly expressed in myelinating OL lineage cells, we used two different OL-specific *cre*-driven knockout mouse lines to study the consequences of *Piezo2* loss-of-function over time. Mice lacking *Piezo2* exhibited decreased body weights and motor deficits in rotarod behavioral testing. Besides, we assessed disrupted compact myelin structure in OL-specific *Piezo2*-deficient ON and spinal cord tissues by electron microscopy (EM), with a pronounced and selective impairment of myelin compaction in small-caliber axons, particularly emerging during aging. In line, differential gene expression (DGE) analysis of snRNA-seq data indicated roles of *Piezo2* in myelination and WM integrity. Also, we proved *PIEZO2* to be downregulated in OLs in normal-appearing WM (NAWM) and peri-plaque WM (PPWM) regions in ON tissue from MS patients, supporting a convergent mechanism of small-caliber fiber vulnerability in human disease. To conclude, our findings identify OL-encoded *Piezo2* as a critical regulator of WM integrity and function, which becomes relevant under chronic inflammatory-demyelinating conditions like MS.

## Results

### *Piezo2* is expressed in OL and fibroblast subtypes in the adult ON

To characterize mRNA expression of Piezo channels in ON cell types under physiological conditions, we first performed snRNA-seq of 24 ON samples from *Piezo2*^*f/f*^ *cre*-negative mice at P120 (*n* = 4 reactions, 6 ONs pooled per reaction). After removing potential doublets and low-quality profiles, 13,173 nuclei remained with an average of 3293 nuclei and 1518 detected genes per nucleus (Supplementary Fig. 1a-d, Supplementary Data 1).

**Fig. 1:**
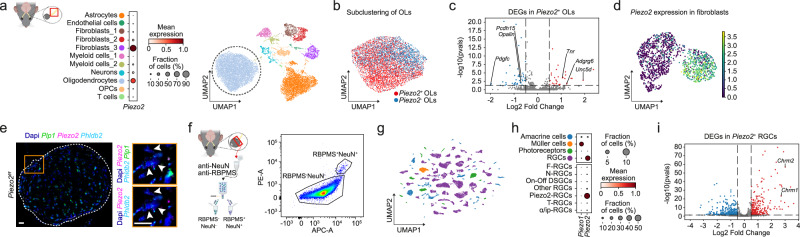
*Piezo2* gene expression in ON and retina of P120 *Piezo2*^*f/f*^ control mice. **a** Dot plot of averaged, z-transformed *Piezo2* expression across cell clusters (*n* = 13,173 nuclei) from snRNA-seq of 24 ON samples (*n* = 4 *Piezo2*^*f/f*^ reactions, 6 samples pooled per reaction, left) with corresponding UMAP visualization (right). **b** UMAP of OL subclustering into *Piezo2*^*+*^ OLs and *Piezo2*- *Piezo2*^*-*^ OLs. **c** Volcano plot of differentially expressed genes comparing *Piezo2*^*+*^ and *Piezo2*^*-*^ OLs (two-tailed Wald test, BH-adjusted). **d** UMAP of *Piezo2* expression in ON fibroblasts. **e** RNA ISH showing *Piezo2* expression in ON fibroblasts (*Phldb2*^*+*^) at meningeal border regions. **f** Schematic of FACS gating strategy for retinal ganglion cell (RGC) isolation (left) and representative flow cytometry plot of RBPMS^*+*^ NeuN^*+*^ RGCs (right). **g** UMAP of retinal cell types (*n* = 13,485 nuclei) from snRNA-seq of 24 retina samples (*n* = 4 *Piezo2*^*f/f*^ reactions, 6 samples pooled per reaction). **h** Dot plots of averaged, z-transformed *Piezo1* and *Piezo2* expression across retinal cell types (top, corresponding to g) and RGC subclusters (bottom). **i**, Volcano plot of differentially expressed genes comparing *Piezo2*^*+*^ and *Piezo2*^*-*^ RGCs (two-tailed Wald test, BH-adjusted). *P < 0.05. Cartoons created in BioRender. Dyckow, J. (2026) https://BioRender.com/z0yinhh.

Using pan-cell type marker genes and published data, we identified 8 major cell types: astrocytes, endothelial cells, fibroblasts, myeloid cells, neurons, oligodendrocytes (OLs), oligodendrocyte precursor cells (OPCs), and T cells (Supplementary Fig. 1e; Supplementary Data 2). The neuronal cluster likely reflects contamination from adjacent brain tissue during dissection. *Piezo1* was primarily expressed in endothelial cells, whereas *Piezo2* revealed highest expression in myelinating OLs and a fibroblast subtype (Fig. 1a, Supplementary Fig. 1e). Within OLs, *Piezo2* expression was heterogeneous (Supplementary Fig. 1f), prompting subclustering into *Piezo2*-expressing (*Piezo2*^*+*^ OLs) and non-expressing OLs (*Piezo2*^*-*^ OLs) (Fig. 1b).

Differential gene expression analysis revealed enrichment of *Tnr* in *Piezo2*^*+*^ OLs, a gene linked to OL maturation and myelin formation^32–34^; while markers of OPCs and early OL stages (e.g., *Pcdh15*, *Pdgfc*^35–38^) were reduced (Fig. 1c; Supplementary Data 1). Further subclustering, by using published data^38^, into myelin-forming OLs (MFOLs) and mature OLs (MOLs) showed strongest *Piezo2* expression in MOLs, particularly in MOL 5-6, representing late-stage adult OLs^38^ (Supplementary Fig. 1g, h; Supplementary Data 3). Consistently, RNA ISH for *Bcas1* showed that most differentiation-committed OPCs and newly-formed OLs did not co-express *Piezo2* (Supplementary Fig. 1i; Supplementary Data 1, 4). In addition, a fibroblast subtype expressed *Piezo2* (Fig. 1a,d), which was spatially confirmed by RNA ISH using the pan-fibroblast marker *Phldb2* (Supplementary Fig. 1j), localizing these cells to ON meningeal border regions (Fig. 1e).

In summary, *Piezo2* marks highly-differentiated, late-stage OLs and a subset of border-associated fibroblasts in the adult mouse ON.

### *Piezo2* is selectively expressed in distinct RGC subtypes in the adult retina

In addition to the ON, we examined *Piezo1/2* gene expression in the retina, whose RGCs project through the ON to the thalamus. RGCs were isolated from 24 retinae of *Piezo2*^*f/f*^ control mice at P120 (*n* = 4 reactions, 6 pooled retinae per reaction) using fluorescence activated cell sorting (FACS) enrichment with antibodies against RBPMS and NeuN (Fig. 1f, Supplementary Fig. 1k). snRNA-seq yielded 13,485 nuclei after quality control, with an average of 3371 nuclei and 2689 detected genes per nucleus (Supplementary Fig. 1l-o, Supplementary Data 1).

Based on established markers and published data^39^, we identified RGCs, amacrine cells, Müller glia, and photoreceptors (Fig. 1g,h, Supplementary Fig. 1p; Supplementary Data 5). *Piezo1* was primarily expressed in Müller glia, whereas *Piezo2* was restricted to specific RGC subclusters (Fig. 1h, Supplementary Fig. 1q). Subclustering identified 4 RGC populations with robust *Piezo2* expression (Supplementary Fig. 1r,s), which were collectively defined as Piezo2-RGCs, distinct from previously described RGC subtypes^40,41^ (Supplementary Fig. 1t). Remaining RGCs were grouped as “Other RGCs*”* (Fig. 1h, Supplementary Fig. 1t; Supplementary Data 6). Next, we analyzed differentially expressed genes (DEGs) comparing *Piezo2*-expressing RGCs (*Piezo2*^*+*^ RGCs) to *Piezo2*-non-expressing RGCs (*Piezo2*^*-*^ RGCs). *Piezo2*^*+*^ RGCs were enriched for *Chrm1* and *Chrm2*, encoding muscarinic acetylcholine receptors^42^ (Fig. 1i; Supplementary Data 1), suggesting that *Piezo2* marks a subset of RGCs associated with cholinergic signaling and potentially specialized retinal circuit function.

### OL-specific *Piezo2* ablation and distinct *Piezo1/Piezo2* expression in OL lineage cells

To investigate the functional role of Piezo2 in OL lineage cells, we generated two different loss-of-function mouse models by crossing *Piezo2*^*f/f*^ mice with either *Olig2-cre* or *Cnp-cre* lines (see methods). Notably, *Olig2-cre*-mediated *Piezo2* loss-of-function mice are heterozygous for *Olig2*; however, previous studies indicate that *Olig2* heterozygosity does not affect myelination^43,44^.

**Fig. 2:**
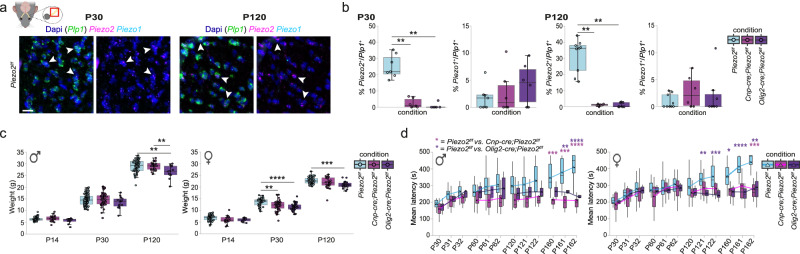
Characterization of OL-specific *Piezo2* loss-of-function mouse models. **a** Representative RNA ISH images of ON cross-sections from *Piezo2*^*f/f*^ mice at P30 and P120 using probes against *Piezo1, Piezo2*, and *Plp1*. Arrowheads indicate *Piezo2*^*+*^*Plp1*^*+*^ OLs. Scale bar: 20 µm. **b** Boxplots showing the percentage of *Piezo2*^+^ (*Piezo2*^+^/*Plp1*^+^) and *Piezo1*^+^ (*Piezo1*^+^/*Plp1*^+^) of all OLs at P30 (left) and P120 (right) in *Piezo2*^*f/f*^ (*n* = 7 (P30), 9 (P120)), *Cnp-cre;Piezo2*^*f/f*^ (*n* = 6 (P30), 6 (P120)), and *Olig2-cre;Piezo2*^*f/f*^ (*n* = 6 (P30), 7 (P120)) mice. Kruskal-Wallis with pairwise Wilcoxon test, Holm-adjusted. **c** Body weight distribution at P14, P30 and P120 in *Piezo2*^*f/f*^, *Cnp-cre;Piezo2*^*f/f*^ and *Olig2-cre;Piezo2*^*f/f*^ mice separated by sex (male, left; female, right). *n* = 10 - 89 mice per sex/genotype/time point (see Methods). Kruskal-Wallis with pairwise Wilcoxon test, Holm-adjusted, or one-way ANOVA with Tukey’s HSD test. Each dot represents one animal. **d** Rotarod performance showing mean latency to fall per day, genotype, and sex (male, left; female, right), assessed over three consecutive days at each time point (P30, P60, P120, P160). Triangles indicate group means. *Piezo2*^*f/f*^: *n* = 19 female (f), 20 male (m); *Cnp-cre;Piezo2*^*f/f*^: *n* = 9 f, 10 m; *Olig2-cre;Piezo2*^*f/f*^*:* *n* = 12 f, 6 m. Two-way ANOVA with Sidak’s multiple comparisons test. Boxplots show median and interquartile range (IQR), with whiskers (error bars) extending to largest and smallest values within 1.5× the IQR. *P < 0.05, **P < 0.01, ***P < 0.0001, ****P < 0.0001. Cartoon created in BioRender. Dyckow, J. (2026) https://BioRender.com/z0yinhh.

RNA ISH for *Piezo1* and *Piezo2* in mouse ON tissue demonstrated a marked reduction of *Piezo2* expression in *Plp1*-expressing (myelinating) OLs in both conditional knockout (cKO) lines compared to *Piezo2*^*f/f*^ controls, confirming efficient OL-specific ablation at 30 days (P30) and 120 days (P120) postnatally (Fig. 2a,b; Supplementary Data 1, 7). In *Piezo2*^*f/f*^ controls*, Piezo2* was expressed in 25% of myelinating OLs at P30, increasing to 32% at P120. In contrast, *Piezo2* was not detected in *Cspg4*-expressing OPCs at either time point (Supplementary Fig. 2a-c; Supplementary Data 1, 7). Conversely, *Piezo1* expression was low in myelinating OLs but enriched in OPCs, with 28% of OPCs expressing *Piezo1* at P30, and 35% at P120 (Fig. 2a,b, Supplementary Fig. 2a-c; Supplementary Data 1, 7). No compensatory upregulation of *Piezo1* was found in either *Piezo2* cKO model (Fig. 2b; Supplementary Data 1, 7).

### *Piezo2* loss-of-function leads to sex-biased motor deficits and reduced body weight

To screen for overt phenotypic abnormalities, we assessed body weight at P14, P30, and P120 in *Piezo2*^*f/f*^ controls as well as *Cnp-cre* and *Olig2-cre Piezo2* cKO mice. In males, reduced body weight was observed only in *Olig2-cre;Piezo2*^*f/f*^ mice at P120 compared to *Piezo2*^*f/f*^ and *Cnp-cre;Piezo2*^*f/f*^ mice (Fig. 2c; Supplementary Data 1, 8). In contrast, females of both cKO lines showed reduced body weight already at P30, with *Olig2-cre;Piezo2*^*f/f*^ mice displaying persistingly decreased body weight through P120 (Fig. 2c; Supplementary Data 1, 8).

Given the association of WM abnormalities with motor deficits^45^, we performed rotarod testing at P30, P60, P120, and P160 to study motor function in both cKO lines. Male cKO mice showed reduced latency to fall only at the latest time point. In contrast, female *Olig2-cre;Piezo2*^*f/f*^ mice exhibited earlier impairment starting at P121 and performed worse than male counterparts, whereas *Cnp-cre;Piezo2*^*f/f*^ females showed milder deficits, emerging only at P162 (Fig. 2d; Supplementary Data 1, 9). Additional hindlimb reflex testing revealed no abnormalities at P30, but *Olig2-cre;Piezo2*^*f/f*^ mice developed impaired reflexes by P120 (Supplementary Fig. 2 d; Supplementary Data 1, 10).

In summary, OL-specific *Piezo2* cKO mice develop reduced body weight and age-dependent motor deficits, with earlier and more pronounced effects in females.

### *Piezo2* loss-of-function does not affect microglial reactivity or OPC differentiation

To assess potential effects of *Piezo2* loss-of-function on microglial reactivity, we quantified microglial abundance based on *Aif1* (encoding for Iba1) expression and assessed reactivity using Iba1 and CD68 immunostaining. No differences in microglial number or activation were observed between cKO and *Piezo2*^*f/f*^ mice at P30 and P120, including *Olig2-cre* (*Olig2-cre*^+/-^) heterozygous animals for IHC (Supplementary Fig. 2e-h; Supplementary Data 1, 11).

To evaluate potential effects on OPC differentiation, we performed *Bcas1* RNA ISH and IHC in ON tissue from *Piezo2* cKO and *Piezo2*^*f/f*^ control mice, again including *Olig2-cre*^+/-^ mice for IHC. No differences were detected between conditions (Supplementary Fig. 2i-k; Supplementary Data 1, 4, 11).

### Ultrastructural imaging indicates that *Piezo2* is required for proper myelin lamination in small-caliber ON axons

**Fig. 3:**
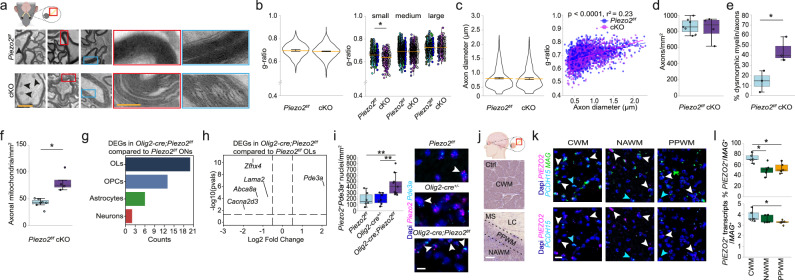
Ultrastructural alterations and transcriptional changes in *Piezo2*-deficient ON tissue and *PIEZO2* downregulation in MS ON tissue. **a** Representative EM images of ONs from P120 *Piezo2*^*f/f*^ and *Olig2-cre;Piezo2*^*f/f*^ (cKO) mice (*n* = 3 per group). Right panels show higher magnification of myelin. Arrowheads indicate mitochondria. Scale bars: 1 µm (overview) and 0.2 µm (insets). **b** G-ratios in ONs across all axon calibers (left; *Piezo2*^*f/f*^: 1840 axons, *n* = 6 mice, median = 0.688; cKO: 1217 axons, *n* = 4 mice, median = 0.694), and stratified by axon diameter (right). Plots show all values with quartiles and median (orange line) based on per-sample means; colors indicate individual mice. Welch Two Sample *t*-test. **c** Axon diameters (*Piezo2*^*f/f*^: *n* = 6 mice, median = 0.807 µm; cKO: *n* = 4 mice, median = 0.792 µm), and correlation analysis. Welch’s two-sample *t*-test (diameter) and nested linear mixed model (correlation). Axonal density (**d**), fraction of axons with dysmorphic myelin (**e**), and axonal mitochondrial counts (**f**) in ONs from *Piezo2*^*f/f*^ (*n* = 7, respectively 3 in e) and cKO mice (*n* = 4, respectively 3 in **e**). Each dot represents the mean of ≥5 images per mouse. Welch’s two-sample *t*-test. Differential gene expression across all ON cell types (**g**) and in OLs (**h**) comparing *Piezo2*^*f/f*^ and cKO samples at P120 (snRNA-seq from 48 ONs; *n* = 4 reactions per condition, 6 pooled samples per reaction). Two-tailed Wald test, BH-adjusted. **i**, Quantification of *Piezo2*^*+*^*Pde3a*^*+*^ nuclei per mm² in P120 ONs from *Piezo2*^*f/f*^ (*n* = 6), *Olig2-cre*^*+/-*^ (*n* = 7), and cKO (*n* = 9) mice, with representative RNA ISH images. Arrowheads indicate double-positive nuclei. Scale bar: 10 µm. One-way ANOVA with Tukey’s HSD test. **j** Representative human ON sections from control (*n* = 7) and MS (*n* = 8) cases. MOG immunohistochemistry identifies lesion core (LC), periplaque white matter (PPWM), and normal-appearing white matter (NAWM) compared to control white matter (CWM). Scale bar: 200 µm. **k** Representative RNA ISH images of *PIEZO2*-expressing *MAG*^*+*^ OLs, co-probed with *PCDH15*, in CWM, NAWM, and PPWM (*n* = 5 per region). White arrowheads indicate *PIEZO2*^*+*^*MAG*^*+*^ OLs; cyan arrowheads indicate *PCDH15*^+^ OPCs. Scale bar: 20 µm. **l** Boxplots showing the percentage of *PIEZO2*^+^ OLs (*PIEZO2*^*+*^*MAG*^*+*^, left) and *PIEZO2* signal counts per *MAG*^*+*^ OL (right). Each dot represents the mean of 6 ROIs per sample. One-way ANOVA with Tukey’s HSD or Kruskal-Wallis test with pairwise Wilcoxon (Holm-corrected). Boxplots show median and interquartile range (IQR), with whiskers (error bars) extending to 1.5× IQR. *P < 0.05, **P < 0.01. Cartoons created in BioRender. Dyckow, J. (2026) https://BioRender.com/z0yinhh.

Given that P120 female *Olig2-cre*;Piezo2^*f/f*^ mice exhibited the strongest motor deficits, we focused on this group for ultrastructural analyses. EM of ONs and spinal cord tissue showed disrupted myelin architecture in cKO mice, characterized by loosely wrapped myelin layers failing to form compact myelin sheaths compared to controls (Fig. 3a, Supplementary Fig. 3a,b). Quantification of g-ratios (axon diameter divided by the axon diameter including the myelin sheath) across all axon calibers showed no differences between cKO and Piezo2^*f/f*^ control mice in either ON (median: 0.688 vs 0.694) or spinal cord (median: 0.653 vs 0.663) (Fig. 3b, Supplementary Fig. 3a, 3b; Supplementary Data 1, 12).

However, given the reported vulnerability of small-caliber axons in MS^30,31^, we stratified axons by diameter. ON WM tissue from *Piezo2*^*f/f*^ control mice predominantly consisted of small-diameter axons (median: 0.807 µm) compared to spinal cord (median: 1.350 µm) (Supplementary Fig. 3c). Subgroup analysis revealed decreased g-ratios specifically in small-diameter ON axons of cKO mice (Fig. 3b, Supplementary Fig. 3d; Supplementary Data 1, 12). In spinal cord WM, no significant differences were observed, although a similar trend was present in small-diameter axons (Supplementary Fig. 3d,e; Supplementary Data 1, 12).

Axon diameters and densities were unchanged in both regions between conditions, indicating that altered myelin structure rather than axonal properties accounts for the observed differences (Fig. 3c,d, Supplementary Fig. 3f; Supplementary Data 1, 12). Consistently, semiquantitative analysis showed an increased proportion of dysmorphic myelin in both ON and spinal cord tissue of cKO mice (Fig. 3e, Supplementary Fig. 3g; Supplementary Data 1, 12). In addition, we found increased mitochondrial density in ON axons of cKO mice, but not in the spinal cord (Fig. 3f, Supplementary Fig. 3h; Supplementary Data 1, 12), suggesting elevated metabolic demand in ONs from cKO mice.

In conclusion, OL-specific *Piezo2* ablation leads to age-dependent myelin lamination defects, predominantly affecting small-caliber axons in the ON, which also exhibits increased mitochondrial content.

### OL-specific *Piezo2* regulates gene expression associated with myelin integrity in ON tissue of adult mice

To investigate the role of OL-encoded *Piezo2* in regulating WM function, we performed DGE analysis on snRNAseq-data from *Olig2-cre;Piezo2*^*f/f*^ cKO and *Piezo2*^*f/f*^ control ON samples at P120. Following integration of cKO and control datasets and cell type clustering (Supplementary Fig. 3i,j), we removed low-quality profiles and obtained 27,952 nuclei (*n* = 4 reactions per genotype; 6 pooled ONs per reaction), with an average of 3494 nuclei and 1467 detected genes per nucleus (Supplementary Fig. 3k-n; Supplementary Data 1, 3). Reclustering identified all major cell types, with an additional OL lineage cluster corresponding to Committed OPCs defined by *Gpr17* and *Sox6* expression (Supplementary Fig. 1e, 3i; Supplementary Data 3).

To assess potential off-target effects of the *Olig2-cre* driver, we performed parallel integration of retinal snRNA-seq datasets from cKO and control mice. We reran cell type clustering after data integration (Supplementary Fig. 3o,p), removed low-quality profiles, and obtained 26,708 nuclei (*n* = 4 reactions per genotype; 6 pooled retinae per reaction), an average of 3339 nuclei and 2585 detected genes per nucleus (Supplementary Fig. 3q-t; Supplementary Data 1, 5). No DEGs were detected in the retina, whereas in the ON, altered gene expression was predominantly restricted to the OL lineage, with the highest number observed in mature OLs (Fig. 3g; Supplementary Data 1). Consistently, *Olig2* expression was largely confined to OL lineage cells in the ON and absent in the retina, supporting the specificity of *Olig2-cre*-mediated *Piezo2* ablation (Supplementary Fig. 3u; Supplementary Data 1, 13). Also, *Cspg4* gene expression in the ON was specific for OPCs, as we used the respective ISH probe for validation experiments (Supplementary Fig. 3v; Supplementary Data 1, 13).

Focusing on OLs, DGE analysis comparing *Olig2-cre;Piezo2*^*f/f*^ OLs to *Piezo2*^*f/f*^ OLs revealed downregulation of *Lama2* in *Piezo2*-deficient OLs, which encodes laminin alpha 2, a regulator of myelination and myelin integrity^46,47^, and *Cacna2d3*, encoding a calcium channel subunit^48^. In contrast, *Pde3a*, encoding a phosphodiesterase regulating intracellular cAMP levels^49^, was upregulated in *Piezo2*-deficient OLs (Fig. 3h; Supplementary Data 1). Increased *Pde3a* expression was validated by RNA ISH (Fig. 3i; Supplementary Data 1, 14), and consistent OL-specific *Piezo2* expression was confirmed using independent probes (Supplementary Fig. 3w). Pathway analysis identified downregulation of epithelial-mesenchymal-transition (EMT)-related processes in *Piezo2*-deficient OLs (Supplementary Fig. 3x; Supplementary Data 1).

Collectively, these data indicate that OL-specific *Piezo2* regulates transcriptional programs linked to myelin integrity and intracellular signaling in the ON in older mice.

### *PIEZO2* downregulation in myelinating OLs and increased OPC densities at ON lesion rims in MS

To assess the relevance of PIEZO2 in human disease, we analyzed postmortem ON tissue from MS and control patients (Supplementary Data 15). Demyelinated lesions and surrounding regions, including NAWM and PPWM – corresponding to lesion rims – were identified by MOG IHC (Fig. 3j).

RNA ISH for *MAG* (encoding for myelin-associated glycoprotein) revealed that 73% of *MAG*-expressing OLs expressed *PIEZO2* in control WM (CWM). In contrast, *PIEZO2* expression was reduced in OLs from NAWM and PPWM in MS tissue, with a progressive decrease in signal intensity per cell toward lesion areas (Fig. 3k,l). The proportion of *MAG*-expressing OLs did not differ between regions.

Probing for *PCDH15*, a marker for human OPCs^50^, showed increased OPC densities in PPWM compared to NAWM (Supplementary Fig. 3y,z; Supplementary Data 1, 16). In parallel, *PIEZO1* was expressed in 81% of OPCs and 46% of OLs in CWM, whereas *PIEZO2* was detected in only 7% of OPCs. Notably, *PIEZO1* expression remained unchanged across NAWM and PPWM in both OPCs and OLs (Supplementary Fig. 3z; Supplementary Data 1, 16).

Taken together, *PIEZO2* expression is progressively reduced in OLs toward MS lesion areas in the human ON, while OPC densities increase at lesion rims and *PIEZO1* expression remains unchanged.

## Discussion

The ability to sense mechanical cues from the microenvironment is essential for proper CNS function. While mechanosensitive Piezo channels are well characterized in the PNS, their role in the CNS, particularly that of Piezo2, has remained largely unexplored. Here, we identify *Piezo2* as a critical regulator of OL function and myelin integrity, with conserved relevance across species, as supported by its downregulation in OLs in human MS lesions.

Our data show that *Piezo2* expression is largely restricted to mature OLs, indicating a role in myelin integrity rather than formation. In contrast, *Piezo1* is predominantly expressed in OPCs, suggesting stage-specific functions of mechanosensitive signaling within the OL lineage. As protein-level detection of Piezo channels remains technically challenging due to limitations of available antibodies^51^, we relied on transcriptomic and RNA-based approaches to define these cell type-specific expression patterns. The differential expression across OL lineage stages supports a model in which Piezo2-mediated mechanotransduction becomes particularly relevant in highly differentiated OL, such as MOL 5-6 subtypes, responsible for maintaining myelin structure over time and exhibiting functions related to repair under demyelinating conditions^38,52^. In line, through transcriptional profiling we found that *Piezo2*-expressing OLs are associated with gene programs linked to maturation, ECM interaction, and myelin stability.

Functionally, OL-specific *Piezo2* ablation resulted in age-dependent reduction in body weight, motor deficits and ultrastructural abnormalities of myelin in both ON and spinal cord WM. In the spinal cord, changes in g-ratios did not reach statistical significance. However, the observed trends suggest signs of impaired myelin integrity along cortico-spinal tracts, too, which may contribute to the age-dependent motor deficits that again likely correlate with reduced body weight. Notably, body weight and motor phenotypes were more pronounced in female mice, indicating a stronger functional impact in this group. At the structural level, alterations were most evident in small-caliber axons of the ON, including disrupted myelin compaction and decreased g-ratios, indicating a fiber size-dependent requirement for Piezo2 and increased vulnerability of small-caliber axons. Given their reduced structural reserve and high metabolic demand, even subtle myelin perturbations are likely to disproportionately affect their function. Mechanistically, our findings support a model in which Piezo2 enables mature OLs to sense and respond to mechanical cues within the myelin sheath—such as interlamellar tension—under physiological conditions, thereby preserving structural myelin integrity. Loss of Piezo2 function may impair this sensing function, leading to progressive myelin instability that becomes more apparent with aging. The delayed onset of deficits suggests that Piezo2 is dispensable for initial myelination but required for long-term WM integrity. While the basis for the more pronounced phenotype in females remains unclear, it may point to a sex-dependent vulnerability of OL function, consistent with known sex differences in autoimmune diseases like MS. We cannot fully exclude that subtle early developmental alterations contribute to the later phenotype. Further, while the use of *Olig2-cre* heterozygous mice may introduce additional effects, prior studies have not identified myelination abnormalities up to 12 weeks of age but potential contributions at later stages remain unresolved^43,44^.

Consistent with a role in supporting the metabolic demands of myelin, we observed increased mitochondrial content in axons of *Piezo2*-deficient WM tracts, indicative of elevated metabolic stress^53,54^. Small-caliber axons rely more strongly on local mitochondrial ATP production and are particularly sensitive to disruptions in myelin integrity^55^. Accordingly, compromised myelin has been shown to trigger a compensatory increase in axonal mitochondrial content^56,57^, reflecting increased metabolic demand under pathological conditions. The predominance of small-caliber fibers in the ON may therefore contribute to the more pronounced phenotype observed in ON compared to spinal cord WM. Of note, smaller axons have been discussed to be more susceptible to injury in MS, too^30,31,58^, a disease for which a positive correlation between myelin decompaction, axonal mitochondrial frequency and decreased g-ratios has been shown^59^. In general, this would be in line with our findings about a relative downregulation of *PIEZO2* in human ON tissue from MS patients. However, these findings remain correlative, and direct causal links between impaired mechanosensation, metabolic stress, and axonal dysfunction require further experimental validation.

We found that *Piezo2* loss-of-function induced transcriptional changes affecting myelin integrity and intracellular signaling in OLs. We observed downregulation of *Lama2* and upregulation of *Pde3a*, regulators with opposing effects on cAMP. *Lama2* promotes cAMP signaling and myelination^60–62^, whereas PDE3A reduces cAMP^49^, consistent with prior evidence that lowered cAMP impairs myelination^63^. In line, reduced *Lama2* expression was described to induce altered myelin compaction^47^. snRNA-seq further indicated alterations in calcium signaling, including downregulation of *Cacna2d3*, suggesting impaired Ca^2+^ homeostasis^48^. Pathway analysis also showed reduced EMT-related signaling, linked to ECM mechanics and cytoskeletal dynamics as shown previously^64,65^. Together, these changes implicate disrupted networks controlling OL differentiation, cytoskeletal organization, and myelin stability. Overall, the data support a role for Piezo2 in linking mechanical cues to intracellular signaling that helps maintain myelin integrity. However, direct downstream mechanisms remain to be defined in future works.

Importantly, our findings extend to human disease. We demonstrate a relative downregulation of *PIEZO2* in MS lesion areas of the ON, particularly at lesion borders, which were also characterized by increased OPC densities, indicative of ongoing repair activity. Given the essential role of Piezo2 in maintaining myelin integrity in small-caliber axons, its downregulation may contribute to the selective vulnerability of these fibers in MS ON tissue. This links OL-intrinsic mechanosensation to a known pathological feature of those lesions, namely the preferential degeneration of small-caliber axons within chronically affected WM, as shown in prior studies of long WM tracts such as the ON^30,59^. However, given the observational nature of the human data, we cannot determine whether *PIEZO2* downregulation represents a causal driver or a consequence of MS lesion pathology. Further, future work needs to clarify if these alterations are more prone to a specific subtype of mature OLs, such as those corresponding to MOL 5-6 in mice, given that specific markers for late-stage human OL subtypes remain limited. While increased OPC densities at lesion rims suggest ongoing repair attempts, this association may be coincidental, and our experimental data do not support a direct role for Piezo2 in early OPC dynamics. Accordingly, impaired Piezo2 signaling may primarily affect myelin integrity rather than early regenerative responses, although effects on repair processes cannot be fully excluded.

In summary, we identify Piezo2 as a mechanosensitive regulator of mature OL function that is required for maintaining myelin integrity, particularly in small-caliber axons during aging. Dysregulation of this pathway in MS suggests that impaired mechanosensation contributes to WM vulnerability and disease progression. These findings provide a conceptual framework linking OL-intrinsic mechanotransduction to a size-selective fiber vulnerability and highlight Piezo2 as a potential therapeutic target to preserve WM integrity in neuroinflammatory diseases. Future work will be required to validate these mechanisms across additional models and to determine whether targeting Piezo2 signaling can restore myelin integrity and improve functional outcomes in demyelinating disease.

## Methods

Experiments and outcome assessments were performed by investigators blinded to experimental conditions. Mice were allocated using stratified randomization, for example by distributing littermates across experimental groups. Handling and processing of mouse and human tissue samples were randomized. A priori sample size calculations for mouse experiments were performed using Noether’s approximation, assuming alpha = 0.05, power of 80%, and an effect size of d = 0.9 (or d = 0.75 for rotarod testing). If not stated otherwise, no samples had to be excluded for analysis.

### Human tissue

Postmortem human ON tissue from MS patients was provided by the UK Multiple Sclerosis Tissue Bank (UKMSTB) at the Imperial College, London via prospective donor scheme following ethical approval by the Wales Research Ethics Committee (18/WA/0238). Control samples were obtained from the UKMSTB and Johns Hopkins University. Samples were obtained from 7 control donors without recognisable neuropathological changes and from 8 donors who were diagnosed with MS. Informed consent was obtained. From human ONs, 16 µm thick tissue sections were obtained with a CM3050S cryostat (Leica Microsystems) and put on superfrost slides (VWR). Further information about the donors can be found in Supplementary Data 15. Sample size was not predetermined using statistical methods. All ethical regulations relevant to human research participants were followed.

### Immunohistochemistry in human ONs

A mouse anti-MOG (clone 8-18C5, 1:1000, Millipore Sigma, RRID AB_1587278) antibody was used to identify demyelinated areas in human ONs. 7 control ON and 8 MS ON samples were stained. Slides were post-fixed in icecold methanol for 5 min. After washing in PBS and blocking in 0.1 M PBS/0.01% Triton X-100/10% goat serum for 30 min at room temperature, the primary antibody was incubated overnight at 4 °C. Slides were washed twice in PBS and incubated with a biotinylated goat anti-mouse secondary antibody (1:500) for 2 h at room temperature. VECTASTAIN Elite ABC-HRP Kit, Peroxidase (PK-6100; Vector Laboratories) was used in combination with ImmPact DAB Substrate Kit, Peroxidase (SK-4105; Vector Laboratories). Nuclei were stained with 50% hematoxylin (ref. 1.05174.1000; Sigma-Aldrich), slides were dehydrated in ethanol, immersed in xylene (Carl Roth) and mounted with Eukitt (ORSAtec; 6272069). Control ONs showed positive DAB signal as expected, three MS samples had to be excluded from further ISH analysis due to no identifiable demyelinated area (one sample) or due to no identifiable NAWM (two samples).

### RNA in situ hybridization

According to the manufacturer’s recommendation, multiplex ISH was performed (RNAscope Multiplex Fluorescent v2 Assay Kit, ACD Biotechne). We used the following manual assay RNAscope probes: *PCDH15* (ref. 525881), *PIEZO1* (ref. 485101), *PIEZO2 (ref. 449951), MAG* (ref. 864691). For mouse tissue, the following probes were used: *Piezo1* (ref. 500511), *Plp1* (ref. 428181), *Cspg4* (ref. 404131), *Pde3a* (ref. 1157721), *Aif1* (ref.319141)*, Bcas1* (ref. 1217861-C1). For *Piezo2*, an exon-specific *Piezo2-E43-E45* probe (ref. 500511), complementary to the *Piezo2* gene sequence removed in the knockout constructs, was used in Figs. 1e, 2a, Supplementary Fig. 2a. Alternatively, a non-exon-specific *Piezo2* probe (ref. 400191) was used in Fig. 3i and Supplementary Fig. 3w. Probes were labeled with TSA Plus Fluorophores (Fluorescein, Cyanine3, Cyanine5; Akoya Biosciences) and nuclei were stained with DAPI. Slides were mounted with ProLong Gold Antifade Mountant (Thermo Fisher Scientific; P36930). For quality control, negative (DapB) probe (ref. 320871) and positive probes (*Polr2a*, *PPIB*, *UBC* for human tissue, ref. 320861 and *Polr2a, Ppib*, *Ubc* for mouse tissue, ref. 320881) were run in parallel.

### Quantification of RNA ISH transcripts

6 human control ONs were stained with *MAG* (channel one probe), *PCDH15* (channel two probe) and *PIEZO1* (channel three probe), while 5 control ONs were stained with *MAG* (channel one probe), *PCDH15* (channel two probe) and *PIEZO2* (channel three probe). Similarly, 5 MS ONs were stained using the same channel combinations. For each control ON, 6 regions of interest (ROIs) (2 central and 4 peripheral ROIs) were manually quantified using ImageJ (Fiji, version 2.14.0/1.54 f). In MS samples, 6 ROIs were quantified in the PPWM as well as in the NAWM. Prior to ISH, the different regions of MS samples were selected based on DAB-MOG staining. The PPWM was defined as the area within a distance of up to 1400 µm next to the lesion rim identified in the DAB-MOG staining, proven also by a decreased *MAG* signal within the ISH images. To determine the average *PIEZO1*/*PIEZO2* expression in *PCDH15*^*+*^/*MAG*^*+*^ cells per sample, the mean value of double-positive cells per sample and region was assessed. A cell was counted positive for a marker if it showed at least two transcript signals associated to the nucleus. For ISH of mouse ONs, whole coronal sections were analyzed from both sexes. For analysis of *Bcas1* expression, 6 *Piezo2*^*f/f*^, 5 *Cnp-cre;Piezo2*^*f/f*^ and 3 *Olig2-cre;Piezo2*^*f/f*^ mice were analyzed at P30, while 5 *Piezo2*^*f/f*^, 4 *Cnp-cre;Piezo2*^*f/f*^ and 4 *Olig2-cre;Piezo2*^*f/f*^ mice were analyzed at P120 (cf. Supplementary Figs. 1i, 2i,j). For analysis of *Piezo1*/*Piezo2* expression in *Plp1*^*+*^/*Cspg4*^*+*^ cells (cf. Fig. 2a,b and Supplementary Fig. 2a-c), samples included 8 *Piezo2*^*f/f*^, 7 *Cnp-cre;Piezo2*^*f/f*^ and 6 *Olig2-cre;Piezo2*^*f/f*^ mice at P30, respectively 9 *Piezo2*^*f/f*^, 6 *Cnp-cre;Piezo2*^*f/f*^ and 7 (for OL analysis), respectively 10 (for OPC analysis) *Olig2-cre;Piezo2*^*f/f*^ mice at P120. At P30, one mouse of each the *Cnp-cre;Piezo2*^*f/f*^ and the *Piezo2*^*f/f*^ condition had to be excluded in either the *Plp1 or Cspg4*-dependent expression analysis because the staining did not work. This exclusion criterion was not established a priori. For analysis of *Aif1*^+^ nuclei, 5 *Piezo2*^*f/f*^, 5 *Cnp-cre;Piezo2*^*f/f*^ and 5 *Olig2-cre;Piezo2*^*f/f*^ mice were analyzed at P30, while 4 *Piezo2*^*f/f*^, 6 *Cnp-cre;Piezo2*^*f/f*^ and 5 *Olig2-cre;Piezo2*^*f/f*^ mice were analyzed at P120 (cf. Supplementary Fig. 2e,f). For analysis of *Piezo2*^*+*^*Pde3a*^*+*^ nuclei, 6 *Piezo2*^*f/f*^, 9 *Olig2-cre;Piezo2*^*f/f*^ and 7 *Olig2-cre* heterozygous (*Olig2-cre*^+/-^) mice were analyzed (cf. Fig. 3g). A cell was counted positive for a marker if it showed at least two transcripts associated with the nucleus.

### Mice

All animal experiments were approved by the local government (Regierungspräsidium Karlsruhe, Germany, G-283/20, G-205/19, I-21/16, I-23/30). An animal-experiment licence containing experimental design and analysis plan was prepared before the study, but is not publicly available. We have complied with all relevant ethical regulations for animal use. Mice were maintained in a specific pathogen-free facility at a temperature range of 20 C to 23 C with a 12/12-h light/dark cycle and had standard diet pellets, water ad libitum and paper tissue as nesting material. Except for male adult mice, they were kept in groups of 4 to 5 animals per cage. For testing of hindlimb reflexes, EM and snRNA-seq analysis, female mice were used. In all other experiments, mice from both sexes were used. The age of the mice is clearly stated in the text and in the figure legends. B6(SJL)-Piezo2tm2.2Apat/J mice^15^ (referred to as *Piezo2*^*f/f*^ mice) were commercially available at The Jackson Laboratory (strain #027720). B6.129-*Olig2tm1.1(cre)Wdr*/J mice, which are knock-in mice having a Cre recombinase inserted into the only exon of the oligodendrocyte transcription factor 2 *Olig2* gene^66^ (referred to as *Olig2-cre* mice), were purchased at The Jackson Laboratory (strain #025567). *Cnp-Cre* knock-in mice were transferred from the Max Planck Institute in Goettingen and had been generated as previously described^67^. *Cnp-cre;Piezo2*^*f/f*^ cKO mice were obtained by crossing *Piezo2*^*f/f*^ mice with *Cnp-Cre* mice. *Olig2-cre;Piezo2*^*f/f*^ cKO mice were obtained by crossing *Piezo2*^*f/f*^ mice with *Olig2-Cre* mice. Breeding was performed in-house and all mice were on a C57BL/6 J background. For information regarding primers and protocols used for genotyping mice by PCR we refer to The Jackson Laboratory and published information^67^. Pre-specified humane endpoint criteria (body weight reduction >20%, severe neurological or respiratory distress) were defined for the entire study. Animals presented in this manuscript did not meet the humane endpoint criteria.

### Rotarod behavioral test

Testing was performed on a rotarod (Ugo Basile) in a room separated from the colony room. Mice could first adapt to the room for 5 min. If male and female mice had to be tested on the same day, males were tested first and brought back to the colony room. Mice were tested on three consecutive trials per day with 10 min breaks in between on three consecutive days for each investigated time point (P30, P60, P120, P160). Each examination day started with one acclimatisation run in which the rotarod was set to 4 rounds per minute and mice had to walk for 1 min. Mice were placed back in their cage for 5 min until the measurements started, in which the rotarod was operated in acceleration mode, consistently accelerating from 4 to 40 rounds per minute over the course of 5 min. The speed at which a mouse fell off the rod was noted down as the latency to fall. Mean latencies per day and mouse were calculated and values were pooled per condition. The same individual mice were tested for the different assessed time points. *n* = 19 female and 20 male *Piezo2*^*f/f*^ mice, 9 female and 10 male *Cnp-cre;Piezo2*^*f/f*^ mice, and 12 female and 6 male *Olig2-cre;Piezo2*^*f/f*^ mice.

### Body weight analysis

Mice were moved to a separate cage on a scale to measure their body weight. Weights were measured at the same time during the day. P14: *Piezo2*^*f/f*^ *n* = 44 females (f), 48 males (m), *Cnp-cre;Piezo2*^*f/f*^ *n* = 19 f, 21 m, *Olig2-cre;Piezo2*^*f/f*^ *n* = 12 f, 10 m. P30 *Piezo2*^*f/f*^ *n* = 46 f, 89 m, C*np-cre;Piezo2*^*f/f*^ *n* = 40 f, 50 m, *Olig2-cre;Piezo2*^*f/f*^ *n* = 14 f, 35 m. P120 *Piezo2*^*f/f*^ *n* = 46 f, 60 m, *Cnp-cre;Piezo2*^*f/f*^ *n* = 27 f, 28 m, *Olig2-cre;Piezo2*^*f/f*^ *n* = 22 f, 12 m.

### Hindlimb reflex

Mice were gently lifted by their tail for a maximum of 12 seconds. When a wild type mouse, or in our case *Piezo2*^*f/f*^ control mouse, is lifted by the tail, it typically extends its hindlimbs outward in a steady 90-120-degree angle^68^. To keep stress as low as possible, this experiment was performed when mice anyways had to be transferred to a new cage for maintenance. Impairment of hindlimb reflex was scored as follows: 0, no signs of impairment, both hindlimbs extended; 1, loss of reflex with flexion of one hindlimb; 1.5, loss of reflex with flexion of both hindlimbs; 2, loss of reflex with clasping toes. Reflex scoring was performed in female mice. P30: *Piezo2*^*f/f*^ *n* = 10, *Cnp-cre;Piezo2*^*f/f*^ *n* = 5, *Olig2-cre;Piezo2*^*f/f*^ *n* = 4. P120: *Piezo2*^*f/f*^ *n* = 10, *Cnp-cre;Piezo2*^*f/f*^ *n* = 9, *Olig2-cre;Piezo2*^*f/f*^ *n* = 9.

### Tissue processing of mouse tissue

Mice were sacrificed and ONs were collected. For IHC and RNA ISH, ONs were put into 4% paraformaldehyde (PFA) for 48 h at 4 °C. After post-fixation, samples were cryoprotected in 30% sucrose in PBS for 48 h at 4 °C and embedded in Tissue-Tek O.C.T. compound (Sakura). Cryosections (16 µm) were collected on superfrost slides (VWR) using a CM3050S cryostat (Leica Microsystems). For EM analysis, ONs were prepared according to Weil and colleagues^69^. In brief, mice were deeply anesthetized by i.p. injection of a ketamine/xylazine mixture and were transcardially perfused with PBS and subsequently with fixative solution (4% PFA, 2.5% glutaraldehyde in phosphate buffer with 0.5% NaCl, pH 7.4). ONs were dissected and and postfixed in the same fixative overnight at 4 °C. Tissue was postfixed with 2% OsO_4_ (Science Services, München, Germany) in 0.1 M phosphate buffer pH 7.3 and embedded in EPON resin (Serva, Heidelberg, Germany) after dehydration with acetone. Ultrathin sections were prepared using a Leica UC7 ultramicrotome (Leica, Wetzlar, Germany) and a 35° diamond knife (Diatome, Biel, Switzerland) and stained with UranylLess™ (Science Services, Munich, Germany). For snRNA-seq analysis, ONs were collected, embedded in Tissue-Tek O.C.T. compound in cryomolds and snap-frozen with isopentane in liquid nitrogen, put onto dry ice and stored at -80 °C. Retinae were dissected in icecold PBS, collected in DNA LoBind tubes (Eppendorf) and directly put onto dry ice and stored at –80 °C.

### Immunohistochemistry in mouse optic nerves

Sections were air dried at room temperature for 15 min, afterwards submerged in icecold methanol for 5 min. They were washed in PBS and blocked in 0.1 M PBS/0.01% Triton X-100/10% goat serum for 1 h at room temperature. Primary antibodies (anti-CD68 (rat monoclonal; clone FA-11; 1:250; Abcam), anti-Iba1 (rabbit polyclonal; 1:500; Wako), anti-Bcas1 (NaBC1 mouse monoclonal; clone unknown; 1:100; Santa Cruz Biotechnology)) were incubated overnight at 4 °C. After washing three times with PBS for 5 min each, 1:500 dilutions of secondary antibodies (goat anti-rabbit, goat anti-mouse, goat anti-rat IgG (H + L); Thermo Fisher Scientific) were incubated for 2 h at room temperature. Slides were washed again three times with PBS for 5 min each, and mounted with Fluoromount-G with DAPI (Thermo Fisher Scientific). For analysis of CD68^+^ Iba1^+^ cell density and Bcas1^+^ cell density at P30, 1 male and 2 female *Piezo2*^*f/f*^ mice, 4 male *Olig2-cre*^*+/-*^ mice, 3 female *Olig2-cre;Piezo2*^*f/f*^ mice, and 1 male and 2 female *Cnp-cre;Piezo2*^*f/f*^ mice were analyzed. For analysis of CD68^+^Iba1^+^ cell density and Bcas1^+^ cell density at P120, 2 male and 2 female *Piezo2*^*f/f*^ mice, 3 male *Olig2-cre*^*+/-*^ mice, 3 male and 2 female *Olig2-cre;Piezo2*^*f/f*^ mice, and 2 male and 1 female *Cnp-cre;Piezo2*^*f/f*^ mice were analyzed.

### Electron microscopy of mouse tissue

EM images were taken with a Zeiss EM912 electron microscope (Carl Zeiss Microscopy GmbH, Oberkochen, Germany) at 5000x or at 8000x using an on-axis 2k CCD camera (TRS, Moorenweis, Germany). Image analysis was performed with ImageJ (Fiji, version 2.0.0-rc-69/1.52 and version 2.14.0/1.54 f). For g-ratio and axon diameter analysis, at least 10 random overview EM pictures at uniform distance were taken in a meandered fashion and analyzed. In ON tissue, g-ratios and axon diameters were analyzed from a total of 1840 axons of 6 *Piezo2*^*f/f*^ mice after one mouse had to be excluded due to insufficient fixation, respectively from 1217 axons of 4 *Olig2-cre;Piezo2*^*f/f*^ mice. For analysis of spinal cord tissue, we focused on the cervical part of the spinal cord, and on the ventrolateral WM. Here, g-ratios and axon diameters were analyzed from a total of 1185 axons of 4 *Piezo2*^*f/f*^ mice, respectively from 1141 axons of 4 *Olig2-cre;Piezo2*^*f/f*^ mice. Axons were analyzed using a grid for random sampling. Axons intercepted by the grid were analyzed. For g-ratio analysis (axon diameter divided by the axon diameter including the myelin sheath), axon diameters were determined from circular areas equivalent to the measured areas, and the mean g-ratio per animal was compared. For analysis of axonal density, mitochondrial density, and semiquantitative analysis of fraction of dysmorphic myelin of all myelinated axons (note rater was blinded to genotype; an axon was selected as dystrophic when the majority of its myelin appeared to be dysmorphic or showed strong whirls, respectively, see Fig. 3a and Supplementary Fig. 3a,b for examples), at least 5 pictures of 3 to 7 *Piezo2*^*f/f*^ and 3 to 4 *Olig2-cre;Piezo2*^*f/f*^ mice were analyzed in the ON, respectively of 4 *Piezo2*^*f/f*^ and 4 *Olig2-cre;Piezo2*^*f/f*^ mice in the spinal cord, and the mean per animal was compared. Further, axons were subgrouped into small, medium, and large-diameter axons using quartile-based thresholds of *Piezo2*^*f/f*^ control axons (thresholds in ONs: 0.5 µm and 1.0 µm; spinal cord: 1.0 µm and 1.6 µm). Specifically, ON axons with a diameter below 0.5 µm (corresponding to the smallest 25% axon diameters) were classified as “small”, ON axons with a diameter between 25% and 75% were classified as “medium” (corresponding to diameters between 0.5 µm and 1.0 µm), and axons with a diameter greater than 1.0 µm (corresponding to the largest 25% axon diameters) were classified as “large”, comparable to a recent study^70^. Accordingly, spinal cord axons with a diameter below 1.0 µm (corresponding to the smallest 25% axon diameters) were classified as “small”, spinal cord axons with a diameter between 25% and 75% were classified as “medium” (corresponding to diameters between 1.0 µm and 1.6 µm), and spinal cord axons with a diameter greater than 1.6 µm (corresponding to the largest 25% axon diameters) were classified as “large”. For each animal, the mean g-ratio was calculated within each diameter subgroup and used as the unit of statistical analysis. EM analyses of ON and spinal cord tissues were not entirely overlapping; 7 mice were shared, while others were unique. The perfusions, imaging and analysis were carried out blinded with regard to the genotype.

### Nuclei isolation from mouse optic nerve tissue samples

Per reaction, 6 mouse ONs were pooled after being cut into 55 µm thick cryosections, and nuclei were isolated according to previously described protocols with minor changes^71^. In short, tissue was homogenized in 2 ml of lysis buffer (0.32 M sucrose, 5 mM CaCl2, 3 mM Mg(Ac)2, 0.1 mM EDTA, 10 mM Tris-HCl, 1 mM dithiothreitol (DTT), 0.5% Triton X-100 in diethyl pyrocarbonate (DEPC)-treated water) supplemented with 0.4 U/µl Protector RNase Inhibitor (Merck, Cat. No. 3335402001), using 15 strokes with a tight pestle and a glass Dounce homogenizer on ice. Afterwards, the lysate was transferred into a 17-ml poly(propylene) ultracentrifuge tube and 3.7 ml of sucrose buffer (1.8 M sucrose, 3 mM Mg(Ac)2, 10 mM Tris-HCl, 1 mM DTT in DEPC-treated water) was pipetted on the bottom, subsequently filled up with lysis buffer and then centrifuged at 107,163.6 *g* for 2.5 h at 4 °C. The supernatant was aspirated and the nuclei pellet was submerged for 20 min on ice in 200 μl of DEPC-treated water-based 1× phosphate-buffered saline, containing 1% BSA and 0.4 U/µl RNase Inhibitor (adapted from published protocols)^72^ and then resuspended. After filtering with 30-μm filters, nuclei were manually counted using a hemocytometer and loaded onto a Chromium X Series controller (10x Genomics, Chromium X Firmware v2.20) aiming for 8000 nuclei per sample. We generated libraries according to the 10x Next GEM 3′ Kit v.3.1 protocol (10x Genomics, CG000204, rev. nos. D/PN-1000121, PN-1000127 and PN-1000213). Recommended quality control was performed and libraries were pooled for subsequent sequencing on an Illumina NovaSeq 6000 system, which was performed at the Institute of Clinical Biology (IKMB, Kiel).

### Nuclei isolation and fluorescence activated nucleus sorting in mouse retina samples

Per reaction, 6 frozen retinae were transferred to a Dounce homogenizer containing 1 ml of icecold NP40 lysis buffer (0.1% NP-40 (Thermo Fisher Scientific, Cat. No. 85124), 10 mM Tris pH 8.0, 1 mM CaCl2, 8 mM MgCl2, 15 mM NaCl, 0.02 U µl–1 DNAse I (Merck Millipore, Cat. No. D4527)) supplemented with 0.2 U µl–1 Ribolock RNase Inhibitor (Thermo Fisher Scientific, Cat. No. EO0382). Homogenization was performed using 20 strokes with the loose pestle, followed by the addition of 1 mL of NP-40 lysis buffer to wash remaining tissue from the pestle. The sample was then homogenized for an additional 20 strokes using the tight pestle. The homogenate was passed through a 100 µm cell strainer with additional NP40 lysis buffer and centrifuged at 500 × g for 5 minutes. To preserve RNA integrity, all buffers, except the washing buffer, were supplemented with 0.16 U µl^–1^ Ribolock RNase inhibitor. Pelleted nuclei were resuspended in staining buffer (Tris base buffer: 10 mM Tris pH 8.0, 1 mM CaCl2, 8 mM MgCl2, 15 mM NaCl, DNAse I 1 U ml^–1^) containing 0.02% Tween and 2% BSA, with primary antibodies targeting NeuN (1:250, Merck Millipore, Cat. No. ABN91) and RBPMS (1:250, abcam, Cat. No. ab194213) for 15 minutes at 4 °C. Nuclei were washed, centrifuged at 500 × g for 5 minutes, and stained with secondary antibodies (anti-chicken 647, 1:500; Jackson ImmunoResearch, Cat. No. 703-605-155 and anti-rabbit PE, 1:200, BioLegend, Cat. No. Poly4064) for 15 minutes at 4 °C. Following another washing step, nuclei were filtered through a 70 µm strainer, resuspended in sorting buffer (Tris base buffer with 2% BSA), and Hoechst (1:2000) was added to visualise nuclei. NeuN + /RBPMS+ nuclei were sorted using a BD FACSDiva device into Ames medium (Sigma-Aldrich, Cat. No. A1420) with 1.5% BSA. Sorted NeuN + /RBPMS+ nuclei were pelleted at 500 × g for 5 minutes at 4 °C, resuspended in approximately 20 µl of 0.04% BSA in PBS, visually inspected, counted in a Neubauer chamber, and adjusted to a concentration of ~600 nuclei µl^–1^. Nuclei were then loaded onto a 10X Chromium Single Cell Chip G (10X Genomics) with a targeted recovery of ~8000 nuclei per channel. Libraries were generated according to the manufacturer´s protocol using the Chromium Single Cell 3’ Reagent Kit version 3.1 (dual index).

### snRNA-seq of mouse tissue

snRNA-seq was performed for 4 reactions per condition. Analyzed conditions were either *Piezo2*^*f/f*^ control ONs, or *Olig2-cre;Piezo2*^*f/f*^ ONs, respectively *Piezo2*^*f/f*^ control retinae, or *Olig2-cre;Piezo2*^*f/f*^ retinae. Per reaction, 6 mouse ONs from 3 female mice were pooled, respectively 6 retinae. The expression count matrices were generated by aligning the sequencing data to the GRCm39-2024-A reference transcriptome with Cell Ranger Count (v.7.0.0). Ambient mRNA was removed using Cellbender (v.0.3.0)^73^.

### Quality control of snRNA-seq data

For downstream processing and analysis scanpy toolkit (v.1.10.4)^74,75^ was used in Python (v.3.11.0). Quality control was performed on each sample separately. Nuclei were filtered based on the number of genes ( < 200 genes) and the percentage of mitochondrial counts ( > 5%). Nuclei identified as outliers were removed from further downstream analysis based on the following criteria: log1p total counts, log1p number of genes (based on counts), percentage of counts in the top 20 genes, and percentage of mitochondrial counts, with thresholds set at 5 median absolute deviations (MAD) and 3 MADs, respectively^76^. Genes expressed in more than 10 nuclei were kept. Finally, the samples were filtered for doublets using DoubletDetection (v.4.2.) (p_thresh=1e-5, voter_thresh=0.5)^77^.

### Integration and cell annotation

To generate a single AnnData object the samples were concatenated and then normalized (target sum = 10000) and log-transformed (log1p). Highly variable genes were identified using the highly_variable_genes function from the scanpy library, and dimensionality reduction was performed using principal component analysis (PCA). scVI (v.1.2.0)^78^ was used for integration to reduce batch-effects between samples. The latent representation obtained from the scVI model was used to construct a neighborhood graph, which facilitated the generation of a uniform manifold approximation and projection (UMAP). The Leiden graph-clustering method^79^ was applied to determine clusters (*Piezo2*^*f/f*^ ONs: resolution = 1.6, *Olig2-cre;Piezo2*^*f/f*^ ONs and *Piezo2*^*f/f*^ ONs combined: resolution = 3, *Piezo2*^*f/f*^ retinae: resolution = 1, *Olig2-cre;Piezo2*^*f/f*^ retinae and *Piezo2*^*f/f*^ retinae combined: resolution = 0.7). Marker genes for each cluster were identified using the rank_genes_groups function, filtering the genes with an adjusted *p*-value < 0.05 and log2 fold change > 0.5. Additionally, differential expression was performed with the scVI model to identify genes that are differentially expressed between the Leiden clusters. These genes, combined with known marker genes^38,39,41^, were used to annotate the clusters manually. Clusters deemed to contain low-quality cells or lacking clear molecular profiles were excluded from further analysis. This process was also used to determine subclusters. We subclustered the OLs from the ON dataset. The MOL subgroup of OLs was further subclustered and annotated, resulting in the subgroups MOL 1-4 and MOL 5-6. The nuclei identified as RGCs were extracted from the *Piezo2*^*f/f*^ control and annotated.

### Differential gene expression analysis

Pseudo-bulk profiles were generated using the get_pseudobulk function from decoupler (v.1.8.0) (min_cells > 10, min_counts > 1000)^80^. For the *Piezo2*^*f/f*^ ONs, OLs were annotated based on their *Piezo2* expression. Genes were then tested for differential expression using PyDESeq2 (v.0.4.12)^81^ and *Piezo2* expression as contrast. A nucleus was considered to be expressing *Piezo2* if the gene count of *Piezo2* per nucleus was greater than zero. Furthermore, we analyzed differentially expressed genes in all cell types comparing *Olig2-cre;Piezo2*^*f/f*^ ONs to *Piezo2*^*f/f*^ ONs. The experimental conditions were used as design factor and contrast. Nuclei expressing mitochondrial, ribosomal or hemoglobin genes were excluded from differential gene expression analysis of the ONs as a further quality control measure. *Olig2* was filtered out by the highly variable genes analysis, but added in manually from the raw data for the analysis of the combined ONs. Nuclei identified as RGCs in the *Piezo2*^*f/f*^ retina samples were annotated depending on their *Piezo2* expression and analysed using *Piezo2* expression as contrast. Additionally, differential gene expression analysis was performed in cell types of the retina dataset comparing *Olig2-cre;Piezo2*^*f/f*^ to *Piezo2*^*f/f*^ samples, using the experimental condition as design factor and contrast. Genes were considered significant if the adjusted *p*-value was less than 0.05 and the absolute log2 fold change greater than 0.5.

### Pseudobulk enrichment analysis

Using decoupler (v.2.1.2)^80^, the pseudobulk enrichment analysis for *Olig2-cre;Piezo2*^*f/f*^ OLs compared to *Piezo2*^*f/f*^ OLs was run and scored using the Univariate Linear Model (ULM) method and the Hallmark gene sets^80^. Pathway scores *p* < 0.05 were considered significant.

### Image acquisition and analysis

All images shown, except for EM images, were taken with a Leica DM6 B Thunder microscope (Leica Application Suite X 3.8.1.26810), equipped with a Leica K5C camera and are z-stack images. Images were acquired at 20x or 40x magnification, were processed using ImageJ (Fiji, version 2.0.0-rc-69/1.52 and version 2.14.0/1.54 f) and exported to vector-based software (Adobe Illustrator version 29.0.1) for figure generation.

### Statistics and reproducibility

Unless otherwise stated, comparisons between two groups were performed using an unpaired *t*-test for normally distributed data and a Mann-Whitney *U* test for non-parametric data. Comparisons among more groups were performed using an ordinary one-way ANOVA with Tukey’s post hoc test for parametric data, or the Kruskal-Wallis test with pairwise Wilcoxon tests and Holm correction for non-parametric data. Differential gene expression analysis of snRNA-seq data was performed using the Wald test. Details on the number of biological replicates and the sample sizes, statistical tests, and *p*-values are provided in the figure legends, the results section, and the Supplementary Data. *P* values are indicated as follows: *P ≤ 0.05, **P ≤ 0.01, ***P ≤ 0.001, ****P ≤ 0.0001. Statistical analyses were performed using R (v4.4.2), GraphPad Prism (v10.0.3), and Python (v3.11.0).

### Supplementary information

Supplementary Data 1-1^6^

Numerical source data for all graphs in the manuscript, cell-type-specific marker genes ON, cell-subtype-specific marker genes OLs, ISH* Bcas1*, cell-type-specific marker genes retina, cell-subtype-specific marker genes RGCs, ISH mouse ON Fig.2, mouse body weights, rotarod, hindlimb reflex, IHC Aif1 and CD68 and Bcas1, EM, violin plots *Olig2* and *Cspg4* expression, ISH *Pde3a*, metadata human ON, ISH human ON.

### Reporting summary

Further information on research design is available in the Nature Portfolio Reporting Summary linked to this article.

## Supplementary information

Supplementary Information

Description of Additional Supplementary files

Supplementary Data 1

Supplementary Data 2-16

Reporting Summary

## Data Availability

Numerical source data for all graphs in the manuscript can be found in Supplementary Data 1 file. The snRNA-seq dataset that support the findings of this study is available at the NCBI Gene Expression Omnibus (GEO), available under the GEO accession GSE304318 and GSE304323. All other data that support the findings of this study are available in the manuscript, in the Supplementary Data, or are available from the corresponding author upon reasonable request.

## Code availability

The scripts used for snRNA-seq analysis in this study are available in Zenodo with the identifier 10.5281/zenodo.15877403^82^.
