# Supplementary material for: Oligodendrocyte Piezo2 is a regulator of age-dependent myelin integrity and dysregulated in multiple sclerosis
Source: Commun Biol. 2026 Jun 20;9:849. doi: 10.1038/s42003-026-10530-3 (PMC13283217; doi:10.1038/s42003-026-10530-3)
Supplement: Supplementary file 1 — Supplementary Information [file 42003_2026_10530_MOESM1_ESM.pdf]

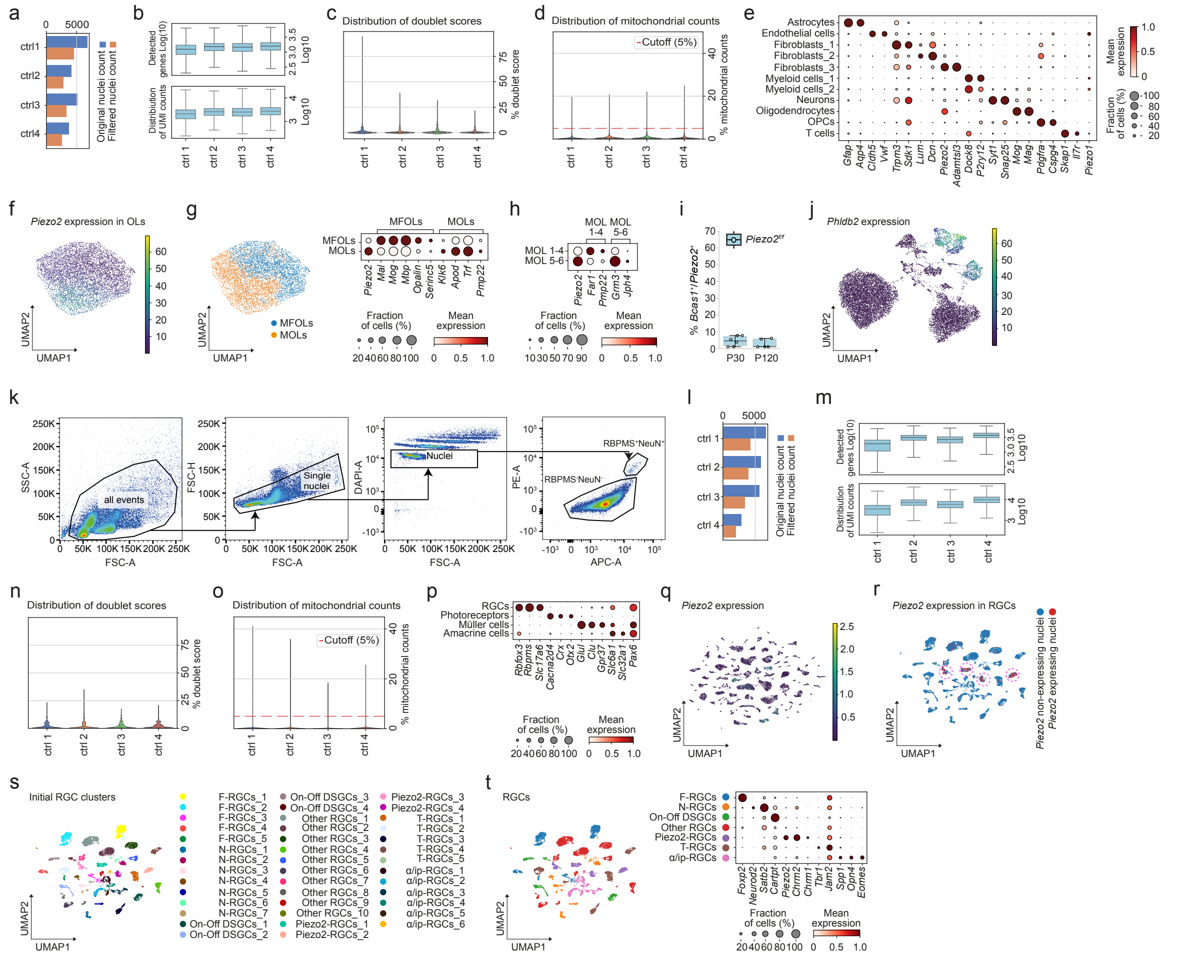

Supplementary Fig. 1. Profiling of *Piezo2* gene expression in ON and retina tissues from P120 *Piezo2$^{f/f}$* control mice.

a - d, Quality control of snRNAseq data of 24 ON samples (n = 4 *Piezo2$^{f/f}$* reactions, 6 samples pooled per reaction, n = 13,173 nuclei) with numbers of nuclei counts before and after filtering (**a**), boxplots showing number of detected genes (top) and distribution of UMI counts (bottom) (**b**), and violin plots showing distribution of doublet scores (**c**) and of mitochondrial counts (**d**). **e,** Dot plot of averaged, z-transformed gene expression of marker genes for identified cell clusters and *Piezo1* gene expression in the snRNA-seq ON dataset. **f,** UMAP visualization of *Piezo2* gene expression distribution in oligodendrocyte (OL) cluster. **g,** UMAP showing subclustering of OLs into myelin-forming OLs (MFOLs) and mature OLs (MOLs, left), according to marker genes (right). **h,** Further subclustering of MOLs into MOL 1-4 and MOL 5-6. **i,** Boxplots visualizing ISH analysis in *Piezo2$^{f/f}$* mouse ONs of *Bcas1*-expressing *Piezo2*$^{+}$ cells (*Bcas1*$^{+}$/*Piezo2*$^{+}$) at P30 (n = 6) and P120 (n = 5). Kruskal-Wallis test. Boxplots with median and interquartile range (IQR) with whiskers (error bars) extending to largest and smallest values within 1.5× the IQR. **j,** UMAP showing *Phldb2* gene expression in all identified fibroblast subclusters. **k,** Representative gating strategy to sort RBPMS^{+} NeuN^{+} RGCs in retina samples. **l - o,** Quality control of snRNAseq data of 24 mouse retinas (n = 4 *Piezo2$^{f/f}$* reactions, 6 retinas pooled per reaction, n = 13,485 nuclei) after flow cytometry sorting for RGCs with numbers of nuclei counts before and after filtering (**l**), numbers of detected genes (top) and distribution of UMI counts (bottom) (**m**), doublet score distribution (**n**)**,** and mitochondrial count distribution (**o**). **p,** Dot plot of averaged, z-transformed gene expression of marker genes for identified cell types in retina dataset after sorting. **q,** UMAP of *Piezo2* gene expression in identified retinal cell clusters. **r,** UMAP of *Piezo2* gene expression in RGC cell clusters. Magenta-colored dashed circles mark clusters identified as Piezo2-RGCs. **s,** UMAP visualization of initial RGC clusters. **t,** UMAP

visualization of identified RGC subclusters (left) with corresponding dot plot (right) showing averaged, z-transformed expression of marker genes.

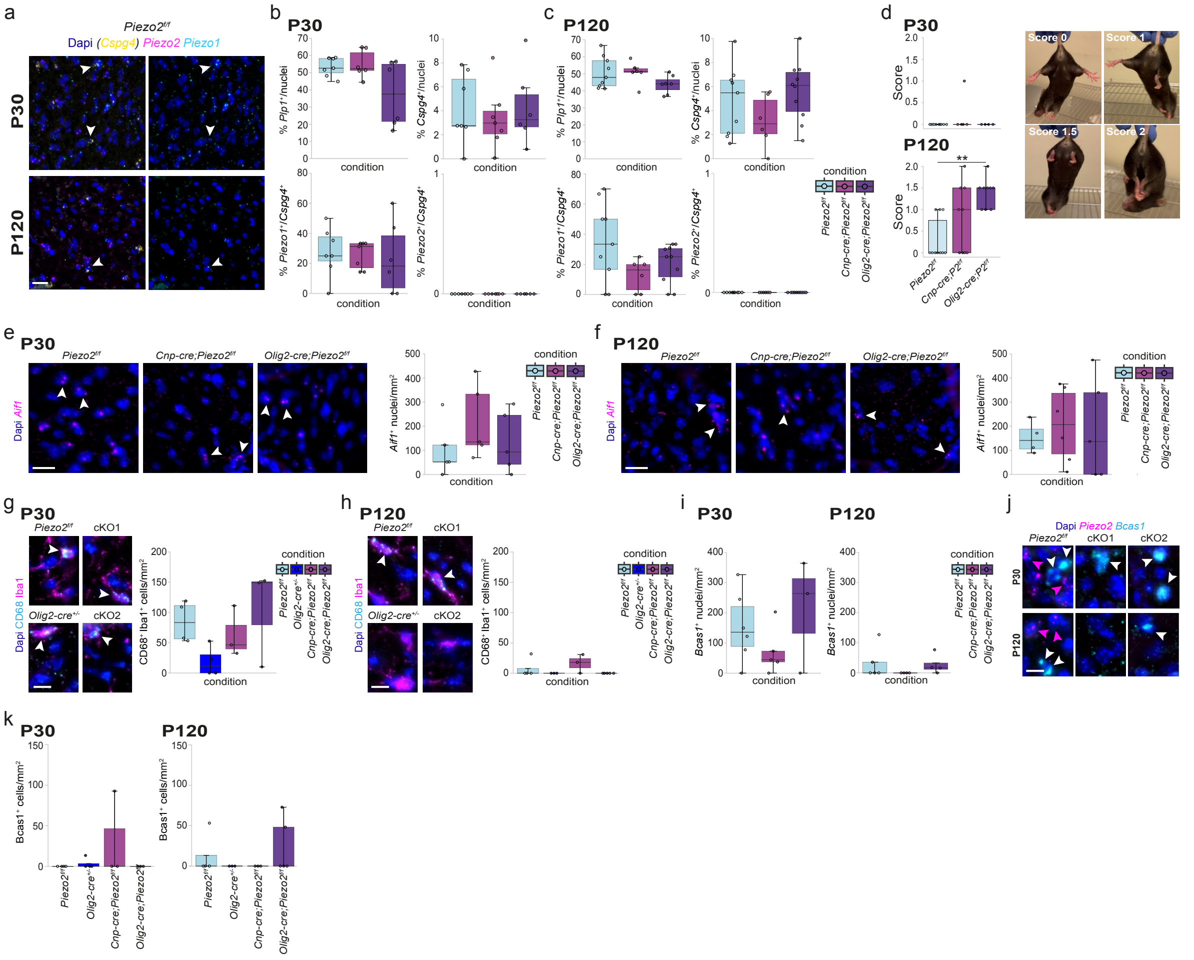

Supplementary Fig. 2. Characterization of microglial reactivity and OPC differentiation in OL-specific *Piezo2* loss-of-function mice.

a, Representative ISH images of *Piezo2*$^{f/f}$ mouse ONs at P30 and P120 using probes against *Piezo1, Piezo2* and *Cspg4*. Arrowheads highlight *Piezo1*$^{+}$*Cspg4*$^{+}$ OPCs. Scale bar: 20µm. **b, c,** Boxplots showing percentages of *Plp1*$^{+}$ OLs and of Cspg4^{+} OPCs of all nuclei, percentages of *Piezo1*$^{+}$ or *Piezo2*$^{+}$ OPCs of all OPCs (*Piezo1*$^{+}$ or *Piezo2*$^{+}$ / *Cspg4*$^{+}$, respectively) at P30 (**b**) and P120 (**c**) in *Piezo2*$^{f/f}$ (n = 7 (P30), 9 (P120)), *Cnp-cre;Piezo2*$^{f/f}$ (n = 7 in *Cspg4*$^{+}$, respectively 6 in *Plp1*$^{+}$ analysis (P30), 6 (P120)), and *Olig2-cre;Piezo2*$^{f/f}$ mice (n = 6 (P30), 10 in *Cspg4*$^{+}$, respectively 7 in *Plp1*$^{+}$ analysis (P120)). One-way ANOVA with Tukey's HSD test or Kruskal-Wallis test. **d,** Boxplots visualizing scoring of hindlimb reflex impairment in *Piezo2*$^{f/f}$ compared to *Cnp-cre;Piezo2*$^{f/f}$ and *Olig2-cre;Piezo2*$^{f/f}$ mice at P30 (*Piezo2*$^{f/f}$ n = 10, *Cnp-cre;Piezo2*$^{f/f}$ n = 5, *Olig2-cre;Piezo2*$^{f/f}$ n = 4), and at P120 (*Piezo2*$^{f/f}$ n = 10, *Cnp-cre;Piezo2*$^{f/f}$ n = 9, *Olig2-cre;Piezo2*$^{f/f}$ n = 9), with representative pictures of P120 mice. Kruskal-Wallis test with pairwise Wilcoxon test, Holm-adjusted. **e - h,** Assessment of microglia. Representative ISH images showing *Aif1*$^{+}$ microglia at P30 (**e**) and P120 (**f**), and IHC images showing CD68^{+} Iba^{+} cells at P30 (**g**) and P120 (**h**). Corresponding quantification in ONs from *Piezo2*$^{f/f}$ (*Aif*: n = 5 (P30), 4 (P120), Iba1: n = 4 (P30, P120)), *Cnp-cre;Piezo2*$^{f/f}$ (cKO1, *Aif*: n = 5 (P30), 6 (P120), Iba1: n = 3 (P30, P120)), and *Olig2-cre;Piezo2*$^{f/f}$ mice (cKO2, *Aif*: n = 5 (P30), 5 (P120), Iba1: n = 3 (P30), 5 (P120)), and additionally for IHC in *Olig2-cre*$^{+/-}$ (n = 3 (P30, P120)) mice. Arrowheads highlight *Aif1*$^{+}$ / CD68^{+} Iba^{+} cells. Scale bar: 20µm (ISH) / 10µm (IHC). One-way ANOVA or Kruskal-Wallis test. **i, j,** ISH analysis of OPC density (*Bcas1*$^{+}$ nuclei/mm^{2}), using an exon-specific (*E43-E45*) *Piezo2* probe and *Bcas1*, in *Piezo2*$^{f/f}$ (n = 6 (P30), 5 (P120)), *Cnp-cre;Piezo2*$^{f/f}$ (cKO1, n = 5 (P30), 4 (P120)), and *Olig2-cre;Piezo2*$^{f/f}$ mice (cKO2, n = 3 (P30), 4 (P120)) (**i**) and representative images. White and magenta arrowheads highlight *Bcas1*$^{+}$ OPCs and *Piezo2*$^{+}$ cells, respectively. Scale

bar: 10μm (**j**). One-way ANOVA or Kruskal-Wallis test. **k,** Quantification of OPC density (Bcas1^{+} cells/mm^{2}) using IHC on ONs from *Piezo2*$^{f/f}$ (n = 4 (P30, P120), *Olig2-cre*$^{+/-}$ (n = 4 (P30), 3 (P120)), *Cnp-cre;Piezo2*$^{f/f}$ (n = 3 (P30, P120), and *Olig2-cre;Piezo2*$^{f/f}$ mice (n = 3 (P30), 5 (P120)). Kruskal-Wallis test. Boxplots with median and interquartile range (IQR) with whiskers (error bars) extending to largest and smallest values within 1.5× the IQR, each dot representing one animal. *P < 0.05, **P < 0.01.

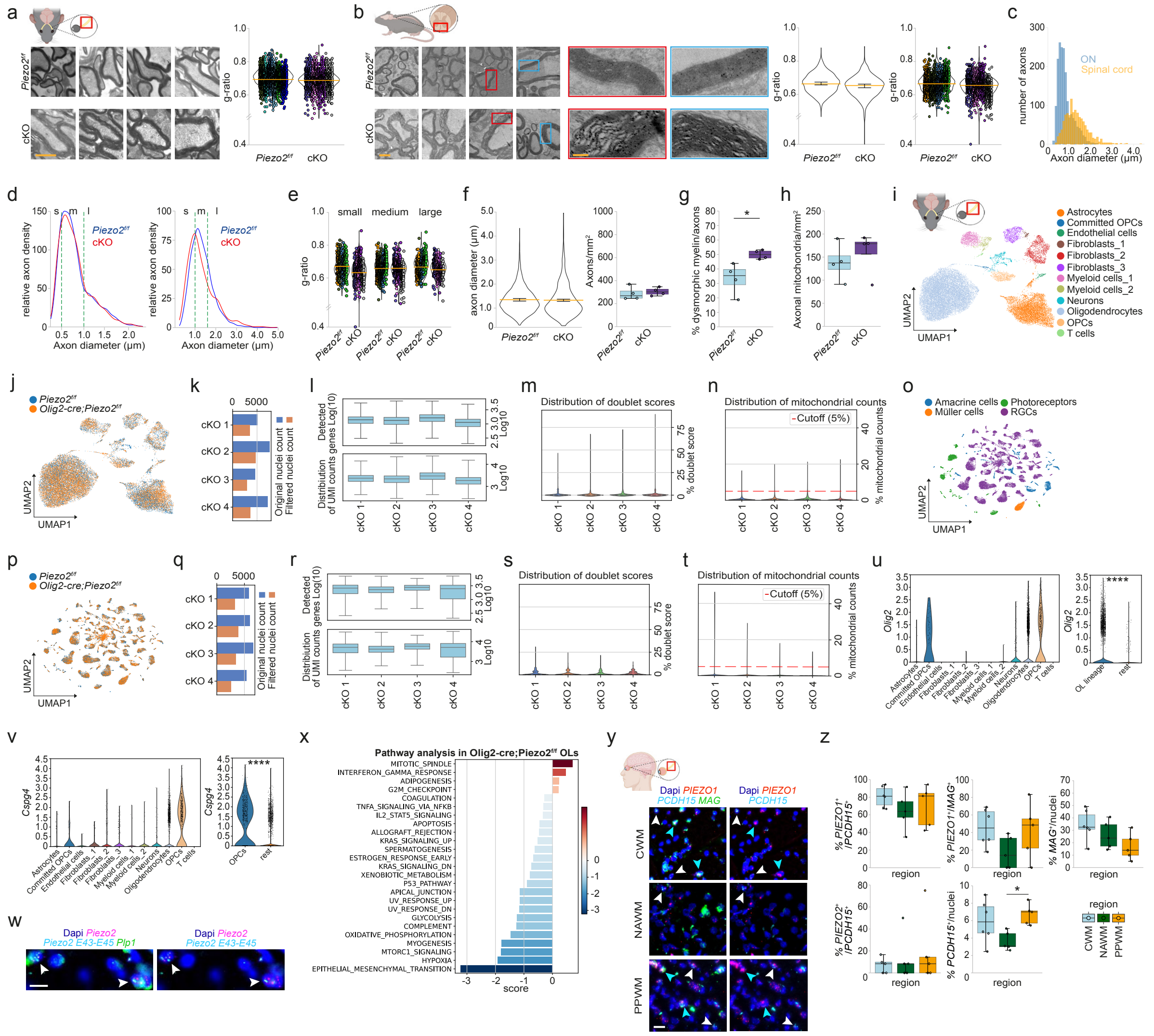

Supplementary Fig. 3. EM imaging of ON and spinal cord tissue, integration of snRNA-seq data from *Olig2-cre;Piezo2$^{f/f}$* ON and retina tissue and ISH for *PIEZO1/PIEZO2* expression in OL lineage cells in human ON tissue.

a, b, ON **(a)** and spinal cord **(b)** data with representative EM pictures from P120 *Piezo2$^{f/f}$* and *Olig2-cre;Piezo2$^{f/f}$* (cKO) mice, and corresponding g-ratios of all axons. ON: 1,840 axons, median (6 *Piezo2$^{f/f}$* mice) = 0.694; 1,217 axons, median (4 cKO mice) = 0.688; spinal cord: 1,185 axons, median (4 *Piezo2$^{f/f}$* mice) = 0.663; 1,141 axons, median (4 cKO mice) = 0.653. Images from two to three mice per condition per region. Scale bars: 1µm (**a, b** overviews) / 0.2µm (insets, **b** center). Plots representing all values, with quartiles and median (orange line) based on means per sample. Separate colors represent individual mice. Welch Two Sample t-test. **c,** Histogram showing axon counts per diameter bin in ON and spinal cord tissues from *Piezo2$^{f/f}$* mice used for EM analysis. **d,** Axon size subgroups of small (s), medium (m), and large (l) diameter axons (dashed lines) defined by the first, second and third and fourth quartiles of *Piezo2$^{f/f}$* axons, in ONs (left) and spinal cords (right). **e,** G-ratio analysis in spinal cord axon subgroups. Separate colors represent individual mice. Welch Two Sample t-test comparing means per sample. Orange line marks median. **f - h,** Axon diameters (**f** left, median of *Piezo2$^{f/f}$* = 1.350; cKO = 1.330; quartiles and median (orange line) based on means per sample), axonal density (**f** right), fraction of axons displaying dysmorphic myelin of all axons (**g**), and axonal mitochondria numbers (**h**), in spinal cords from *Piezo2$^{f/f}$* and cKO mice. Each dot represents the mean of at least 5 analyzed images per mouse. Welch Two Sample t-test or Mann-Whitney U test. **i, j,** UMAP visualization of cell clusters (n = 27,952 nuclei) (**i**) and condition (**j**) in integrated snRNA-seq dataset of 48 ONs (n = 4 reactions per

condition, 6 pooled ONs per reaction). **k - n,** Quality control of snRNA-seq ON data from *Olig2-cre;Piezo2*$^{f/f}$ mice, plots showing nuclei counts before and after filtering (**k**), number of detected genes (top) and distribution of Unique Molecular Identifier (UMI) counts (bottom panel) (**l**), distribution of doublet scores (**m**) and mitochondrial counts (**n**). **o, p,** UMAP visualization of cell clusters (n = 26,708 nuclei) (**o**) and condition (**p**) in integrated snRNA-seq dataset of 48 retinae (n = 4 reactions per condition, 6 pooled retinae per reaction). **q - t,** Quality control of snRNA-seq retina data from *Olig2-cre;Piezo2*$^{f/f}$ mice, plots showing nuclei counts before and after filtering (**q**), number of detected genes (top) and distribution of Unique Molecular Identifier (UMI) counts (bottom panel) (**r**), distribution of doublet scores (**s**) and mitochondrial counts (**t**). **u,** Violin plots showing *Olig2* expression in ON cell clusters of both conditions (left), and compared between OL lineage (containing OPCs, Committed OPCs and OLs) and residual cell types (right). **v,** Violin plots showing *Cspg4* expression across cell clusters (left) and compared between OPCs and residual cell types (right). Two-tailed Mann-Whitney U test, BH-adjusted. **w,** Representative ISH image of a murine ON (P120 *Piezo2*$^{f/f}$) using an exon-specific (*E43-E45*) and a non-exon-specific *Piezo2* probe, with *Plp1* for OLs. Arrowheads highlight *Piezo2 E43-E45*$^+$*Piezo2*$^+$*Plp1*$^+$ OLs. Scale bar: 10µm. **x,** Pathway analysis comparing *Olig2-cre;Piezo2*$^{f/f}$ OLs to *Piezo2*$^{f/f}$ OLs. Univariate Linear Model. **y,** Representative ISH images of *PIEZO1*-expressing *PCDH15*$^+$ OPCs, co-stained for *MAG* in CWM (first row, n = 6), NAWM and PPWM (second and third row, respectively, n = 5 each). White and cyan arrowheads highlight *PIEZO1*$^+$*PCDH15*$^+$ OPCs and *PIEZO1*$^+$*MAG*$^+$ OLs, respectively. Scale bar: 20µm. **z,** Boxplots showing percentage of *PIEZO1*$^+$ OPCs of *PCDH15*$^+$ OPCs (top left), percentage of *PIEZO1*$^+$ OLs of *MAG*$^+$ OLs (top center), percentage of *MAG*$^+$ OLs per nuclei (top right), percentage of *PIEZO2*$^+$ OPCs of *PCDH15*$^+$ OPCs (bottom left) and percentage of *PCDH15*$^+$ OPCs per nuclei (bottom center) in CWM, NAWM, and PPWM. Each dot represents the mean of 6 analyzed ROIs per sample. Boxplots with median and interquartile range (IQR) with whiskers (error bars) extending to largest and smallest values within 1.5× the IQR. Ordinary one-way ANOVA test or Kruskal-Wallis test. *$P < 0.05$, **$P < 0.01$, ***$P < 0.0001$, ****$P < 0.0001$. Cartoons created in BioRender. Dyckow, J. (2026) https://BioRender.com/z0yinhh.
