# Supplementary material for: Oligodendrocyte Piezo2 is a regulator of age-dependent myelin integrity and dysregulated in multiple sclerosis
Source: Commun Biol. 2026 Jun 20;9:849. doi: 10.1038/s42003-026-10530-3 (PMC13283217; doi:10.1038/s42003-026-10530-3)
Supplement: Supplementary file 2 — Description of Additional Supplementary files [file 42003_2026_10530_MOESM2_ESM.pdf]

Description of Additional Supplementary files

Supplementary Data 1

Numerical source data for graphs in the manuscript.

Supplementary Data 2

Cell-type-specific marker genes for mouse optic nerves.

Supplementary Data 3

Marker genes for oligodendrocyte subtypes.

Supplementary Data 4

In situ hybridization analysis of Bcas1 in mouse optic nerves.

Supplementary Data 5

Cell-type-specific marker genes for mouse retinae.

Supplementary Data 6

Marker genes for retinal ganglion cell (RGC) subtypes.

Supplementary Data 7

In situ hybridization analysis of Piezo1/Piezo2 gene expression in Cspg4/Plp1 expressing cells in mouse optic nerves.

Supplementary Data 8

Analysis of mouse body weight.

Supplementary Data 9

Rotarod analysis in mice.

Supplementary Data 10

Analysis of hindlimb reflex in mice.

Supplementary Data 11

Immunohistochemical analysis of Aif1, CD68, and Bcas1.

Supplementary Data 12

Electron microscopy (EM) analysis of murine optic nerve and spinal cord tissue.

Supplementary Data 13

Olig2 and Cspg4 gene expression in optic nerves integrated from Piezo2f/f and Olig2-cre;Piezo2f/f mice.

Supplementary Data 14

In situ hybridization analysis of Piezo2+ Pde3a+ in mouse optic nerves.

Supplementary Data 15

Metadata for human optic nerve tissue.

Supplementary Data 16

In situ hybridization analysis in human optic nerve tissue.
