# Supplementary material for: Oligodendrocyte Piezo2 is a regulator of age-dependent myelin integrity and dysregulated in multiple sclerosis
Source: Commun Biol. 2026 Jun 20;9:849. doi: 10.1038/s42003-026-10530-3 (PMC13283217; doi:10.1038/s42003-026-10530-3)
Supplement: Supplementary file 5 — Reporting Summary [file 42003_2026_10530_MOESM5_ESM.pdf]

nature portfolio

Corresponding author(s): Lucas Schirmer

Last updated by author(s): Jun 5, 2026

Reporting Summary

Nature Portfolio wishes to improve the reproducibility of the work that we publish. This form provides structure for consistency and transparency in reporting. For further information on Nature Portfolio policies, see our Editorial Policies and the Editorial Policy Checklist.

Statistics

For all statistical analyses, confirm that the following items are present in the figure legend, table legend, main text, or Methods section.

n/a	Confirmed	
☐	☒	The exact sample size (*n*) for each experimental group/condition, given as a discrete number and unit of measurement
☐	☒	A statement on whether measurements were taken from distinct samples or whether the same sample was measured repeatedly
☐	☒	The statistical test(s) used AND whether they are one- or two-sided<br>*Only common tests should be described solely by name; describe more complex techniques in the Methods section.*
☐	☒	A description of all covariates tested
☐	☒	A description of any assumptions or corrections, such as tests of normality and adjustment for multiple comparisons
☐	☒	A full description of the statistical parameters including central tendency (e.g. means) or other basic estimates (e.g. regression coefficient) AND variation (e.g. standard deviation) or associated estimates of uncertainty (e.g. confidence intervals)
☐	☒	For null hypothesis testing, the test statistic (e.g. *F*, *t*, *r*) with confidence intervals, effect sizes, degrees of freedom and *P* value noted<br>*Give P values as exact values whenever suitable.*
☒	☐	For Bayesian analysis, information on the choice of priors and Markov chain Monte Carlo settings
☐	☒	For hierarchical and complex designs, identification of the appropriate level for tests and full reporting of outcomes
☐	☒	Estimates of effect sizes (e.g. Cohen's *d*, Pearson's *r*), indicating how they were calculated

Our web collection on statistics for biologists contains articles on many of the points above.

Software and code

Policy information about availability of computer code

Data collection	Leica Application Suite X 3.8.1.26810, ImageJ (Fiji, version 2.0.0-rc-69/1.52 and version 2.14.0/1.54f), Chromium X Firmware v2.20.
Data analysis	ImageJ (Fiji, version 2.14.0/1.54f), R software version 4.4.2, Prism software (version 10.0.3; GraphPad Software), Python version 3.11.0, Adobe Illustrator version 29.0.1, Cell Ranger Count version 7.0.0, Cellbender version 0.3.0, link to bioninformatic code: https://doi.org/10.5281/zenodo.15877404

For manuscripts utilizing custom algorithms or software that are central to the research but not yet described in published literature, software must be made available to editors and reviewers. We strongly encourage code deposition in a community repository (e.g. GitHub). See the Nature Portfolio guidelines for submitting code & software for further information.

Data

Policy information about availability of data

All manuscripts must include a data availability statement. This statement should provide the following information, where applicable:

- Accession codes, unique identifiers, or web links for publicly available datasets
- A description of any restrictions on data availability
- For clinical datasets or third party data, please ensure that the statement adheres to our policy

Scripts and bioinformatic data are publicly available. All other data supporting the findings of this study are available within the paper and its Supplementary Data. Numerical source data for graphs in the manuscript can be found in supplementary data 1 file.

Research involving human participants, their data, or biological material

Policy information about studies with human participants or human data. See also policy information about sex, gender (identity/presentation), and sexual orientation and race, ethnicity and racism.

Reporting on sex and gender	Sex was determined based on self-reporting. Data was not disaggregated in terms of sex due to insufficient sample size for meaningful analysis.
Reporting on race, ethnicity, or other socially relevant groupings	Information on race, ethnicity or other socially relevant groupings was not available.
Population characteristics	In total, postmortem optic nerve tissue from 15 samples (7 controls and 8 MS donors) were included for histological validation, and 12 samples (7 controls and 5 MS donors) were included for in situ hybridization analysis, from which one control sample was used only for control stainings. Patients ranged between 17 and 80 years old with a mean of 54,8 years of age.
Recruitment	Postmortem human ON tissue from MS patients was provided by the UK Multiple Sclerosis Tissue Bank (UKMSTB) at the Imperial College, London. Control samples were obtained from the UKMSTB and Johns Hopkins University.
Ethics oversight	MS and control tissue was obtained following fully informed consent by the donors from prospective donor programs following ethical approval by the Wales Research Ethics Committtee (18/WA/0238).

Note that full information on the approval of the study protocol must also be provided in the manuscript.

Field-specific reporting

Please select the one below that is the best fit for your research. If you are not sure, read the appropriate sections before making your selection.

☒ Life sciences ☐ Behavioural & social sciences ☐ Ecological, evolutionary & environmental sciences

For a reference copy of the document with all sections, see nature.com/documents/nr-reporting-summary-flat.pdf

Life sciences study design

All studies must disclose on these points even when the disclosure is negative.

Sample size	For human data, sample size was determined based on tissue availability and quality parameters described in methods. No sample size calculation was performed. The amount of samples selected is enough to perform statistical analysis and prove that the findings are not due to one sample, but we can see the same changes across conditions. For mouse data, a priori sample size calculation was performed using Noether's approximation, assuming alpha = 0.05, power of 80 %, and an effect size of d = 0.9, respectively of 0.75 for rotarod testing.
Data exclusions	Three MS samples had to be excluded from further in situ hybridization analysis due to no identifiable demyelinated area (one sample) or due to no identifiable NAWM region (two samples). In mouse experiments, for in situ hybridization analysis at P30 one mouse of each the Cnp-cre;Piezo2f/f and the Piezo2f/f condition had to be excluded in either the Plp1 or Cspg4-dependent expression analysis because the staining did not work. For g-ratio analysis of ON tissue, one Piezo2f/f mouse had to be excluded due to insufficient fixation.
Replication	Our study did not include a replication study of our findings in independent samples. Advanced computational tools for analyzing snRNA-seq data help manage technical variability, reducing the need for formal replicates and from technical batch effects.
Randomization	Stratified randomization of mice was taken into consideration by distributing littermates across assessed conditions. Handling and processing mouse and human tissue samples in terms of condition was randomized.
Blinding	Selection of human samples for in situ hybridization was not blinded because it was necessary to assess the presence of NAWM, PPWM and lesion core before further analysis. For snRNA-seq analysis, the leiden unsupervised clustering algorithm (Scanpy version 1.10.4) was used. All other experiments were performed blinded, after mice of a certain genotype and age have been assessed to experimental groups.

Reporting for specific materials, systems and methods

We require information from authors about some types of materials, experimental systems and methods used in many studies. Here, indicate whether each material, system or method listed is relevant to your study. If you are not sure if a list item applies to your research, read the appropriate section before selecting a response.

Materials & experimental systems

n/a	Involved in the study
☐	☒ Antibodies
☒	☐ Eukaryotic cell lines
☒	☐ Palaeontology and archaeology
☐	☒ Animals and other organisms
☒	☐ Clinical data
☒	☐ Dual use research of concern
☒	☐ Plants

Methods

n/a	Involved in the study
☒	☐ ChIP-seq
☐	☒ Flow cytometry
☒	☐ MRI-based neuroimaging

Antibodies

Antibodies used	Primary antibodies: mouse anti-MOG (clone 8-18C5, 1:1000, Millipore Sigma, RRID AB_1587278), rat anti-CD68 (monoclonal; clone FA-11; 1:250; Abcam), rabbit anti-Iba1 (polyclonal; 1:500; Wako), anti-Bcas1 (NaBC1 mouse monoclonal; clone unknown; 1:100; Santa Cruz Biotechnology), anti-NeuN (1:250, Merck Millipore, Cat. No. ABN91), anti-RBPMS (1:250, abcam, Cat. No. ab194213).<br><br>Secondary antibodies: biotinylated goat anti-mouse (1:500, Thermo Fisher Scientific, 62-6540); goat anti-rat, goat anti-mouse, goat anti-rabbit IgG (H+L) (Thermo Fisher Scientific; 1:500; A-11006); anti-chicken 647 (1:500, Jackson ImmunoResearch, 703-605-155); anti-rabbit PE ((1:200, BioLegend, Poly4064).
Validation	All listed antibodies are commercially available. The same antibodies and lot have been successfully used in recent peer-reviewed articles, for example (Kapell et al. Neuron-oligodendrocyte potassium shuttling at nodes of Ranvier protects against inflammatory demyelination. J Clin Invest 133, (2023)).

Animals and other research organisms

Policy information about studies involving animals; ARRIVE guidelines recommended for reporting animal research, and Sex and Gender in Research

Laboratory animals	This study involved laboratory mice on a C57BL/6J background. Mice from strain B6(SJL)-Piezo2tm2.2Apat/J were commercially available at The Jackson Laboratory as strain #027720. Mice from strain B6.129-Olig2tm1.1(cre)Wdr/J were commercially available at The Jackson Laboratory as strain #025567. Cnp-cre knock-in mice were transferred from the Max Planck Institute in Goettingen and had been generated as previously described (Lappe-Siefke, C. et al. Disruption of Cnp1 uncouples oligodendroglial functions in axonal support and myelination. Nat Genet 33, 366–374 (2003)). Different time points were analyzed up to P14, P30, P120 and P160 depending on the experiment and the age of the assessed mice is clearly stated in the text and in the figure legends. Mice were maintained in a specific pathogen-free facility at a temperature range of 20C to 23C with a 12/12-h light/dark cycle and had standard diet pellets, water ad libitum and paper tissue as nesting material. Except for male adult mice, they were kept in groups of four to five animals per cage. Breeding was performed in-house.
Wild animals	This study did not involve wild animals.
Reporting on sex	Initial screening of mouse optic nerves by ISH and IHC was performed in both sexes. For body weight analysis and rotarod testing, sex data was disaggregated. As female mice showed stronger motor deficits in rotarod testing, subsequent analysis of hindlimb reflexes, EM and snRNA-seq analysis was carried out in female mice.
Field-collected samples	The study did not involve samples collected from the field.
Ethics oversight	The local government (Regierungspräsidium Karlsruhe, Germany) approved the protocol used for working with mice in this study.

Note that full information on the approval of the study protocol must also be provided in the manuscript.

Plants

Seed stocks

Report on the source of all seed stocks or other plant material used. If applicable, state the seed stock centre and catalogue number. If plant specimens were collected from the field, describe the collection location, date and sampling procedures.

Novel plant genotypes

Describe the methods by which all novel plant genotypes were produced. This includes those generated by transgenic approaches, gene editing, chemical/radiation-based mutagenesis and hybridization. For transgenic lines, describe the transformation method, the number of independent lines analyzed and the generation upon which experiments were performed. For gene-edited lines, describe the editor used, the endogenous sequence targeted for editing, the targeting guide RNA sequence (if applicable) and how the editor was applied.

Authentication

Describe any authentication procedures for each seed stock used or novel genotype generated. Describe any experiments used to assess the effect of a mutation and, where applicable, how potential secondary effects (e.g. second site T-DNA insertions, mosiacism, off-target gene editing) were examined.

Flow Cytometry

Plots

Confirm that:

☒ The axis labels state the marker and fluorochrome used (e.g. CD4-FITC).

☒ The axis scales are clearly visible. Include numbers along axes only for bottom left plot of group (a 'group' is an analysis of identical markers).

☒ All plots are contour plots with outliers or pseudocolor plots.

☒ A numerical value for number of cells or percentage (with statistics) is provided.

Methodology

Sample preparation

Frozen mouse retina samples were transferred to a Dounce homogenizer containing 1 ml of ice-cold NP40 lysis buffer (0.1% NP-40 (Thermo Fisher Scientific, Cat. No. 85124), 10 mM Tris pH 8.0, 1 mM CaCl2, 8 mM MgCl2, 15 mM NaCl, 0.02 U μl–1 DNAse I (Merck Millipore, Cat. No. D4527)) supplemented with 0.2 U μl–1 Ribolock RNAse Inhibitor (Thermo Fisher Scientific, Cat. No. EO0382). Homogenization was performed using 20 strokes with the loose pestle, followed by the addition of 1 mL of NP-40 lysis buffer to wash remaining tissue from the pestle. The sample was then homogenized for an additional 20 strokes using the tight pestle. The homogenate was passed through a 100 μm cell strainer with additional NP40 lysis buffer and centrifuged at 500 × g for 5 minutes. To preserve RNA integrity, all buffers, except the washing buffer, were supplemented with 0.16 U μl–1 Ribolock RNAse inhibitor. Pelleted nuclei were resuspended in staining buffer (Tris base buffer: 10 mM Tris pH 8.0, 1 mM CaCl2, 8 mM MgCl2, 15 mM NaCl, DNAse I 1 U ml–1) containing 0.02% Tween and 2% BSA, with primary antibodies targeting NeuN (1:250, Merck Millipore, Cat. No. ABN91) and RBPMS (1:250, abcam, Cat. No. ab194213) for 15 minutes at 4 °C. Nuclei were washed, centrifuged at 500 × g for 5 minutes, and stained with secondary antibodies (anti-chicken 647 and anti-rabbit PE) for 15 minutes at 4 °C. Following another washing step, nuclei were filtered through a 70 μm strainer, resuspended in sorting buffer (Tris base buffer with 2% BSA), and Hoechst (1:2000) was added to visualise nuclei. NeuN+/RBPMS+ nuclei were sorted.

Instrument

BD FACSAria III

Software

BD FACSDiva Software

Cell population abundance

Single nucleus RNA-sequencing of post-sort fractions showed a distribution of 72.42% RGCs, 14.23% amacrine cells, 9.97% photoreceptors and 3.39% Müller glia cells.

Gating strategy

The preliminary gating of the starting cell population was performed on a FSC-A vs. SSC-A plot, excluding cell debris (low FSC/SSC) and outliers (high SSC/FSC). "Positive" and "negative" populations werde defined on a PE-A vs. APC-A plot, in which the double-positive (RBPMS+/NeuN+) nuclei of interest accumulated in the upper-right quadrant and were identified as positive.

☒ Tick this box to confirm that a figure exemplifying the gating strategy is provided in the Supplementary Information.
